# Transcriptional Regulation Is a Major Controller of Cell Cycle Transition Dynamics

**DOI:** 10.1371/journal.pone.0029716

**Published:** 2012-01-06

**Authors:** Alessandro Romanel, Lars Juhl Jensen, Luca Cardelli, Attila Csikász-Nagy

**Affiliations:** 1 The Microsoft Research-University of Trento Centre for Computational and Systems Biology, Trento, Italy; 2 Novo Nordisk Foundation Center for Protein Research, University of Copenhagen, Copenhagen, Denmark; 3 Microsoft Research Cambridge, Cambridge, United Kingdom; National Cancer Institute, United States of America

## Abstract

DNA replication, mitosis and mitotic exit are critical transitions of the cell cycle which normally occur only once per cycle. A universal control mechanism was proposed for the regulation of mitotic entry in which Cdk helps its own activation through two positive feedback loops. Recent discoveries in various organisms showed the importance of positive feedbacks in other transitions as well. Here we investigate if a universal control system with transcriptional regulation(s) and post-translational positive feedback(s) can be proposed for the regulation of all cell cycle transitions. Through computational modeling, we analyze the transition dynamics in all possible combinations of transcriptional and post-translational regulations. We find that some combinations lead to ‘sloppy’ transitions, while others give very precise control. The periodic transcriptional regulation through the activator or the inhibitor leads to radically different dynamics. Experimental evidence shows that in cell cycle transitions of organisms investigated for cell cycle dependent periodic transcription, only the inhibitor OR the activator is under cyclic control and never both of them. Based on these observations, we propose two transcriptional control modes of cell cycle regulation that either STOP or let the cycle GO in case of a transcriptional failure. We discuss the biological relevance of such differences.

## Introduction

The cell division cycle is controlled by a complex regulatory network that ensures the proper order and timing of DNA replication, mitosis and division of cells [1]. The core regulators are cyclin dependent kinases (Cdks) that periodically get activated by cyclins. These cyclins and many other cell cycle regulators are under periodic transcriptional regulation [2], and it has been recently shown that these transcriptional waves continue even if cyclins are perturbed [3]. Still, the critical cell cycle transitions of G1/S, G2/M and M/G1 are all controlled by significant changes in Cdk activity and only one Cdk/cyclin complex is enough to drive the cell cycle [4]. It was proposed that cell cycle transitions are controlled by positive feedback loops [5], [6] making the transitions work as irreversible switches [7], [8]. The G2/M transition has been extensively studied in frog eggs and in fission yeast cells and a picture emerged, in which Cdk activity is inhibited by Wee1 and activated by Cdc25 [9]. It has been shown that Cdk can post-translationally activate its activator, Cdc25 and inhibit its inhibitor, Wee1 [10]. Both of these effects create positive feedback loops that can lead to bistability - when the system can be in either one of two distinct steady states. Such bistability has been observed experimentally by showing a higher critical cyclin level to activate Cdk than the cyclin level needed to keep Cdk active, proving the system is bistable between the two critical cyclin levels [11], [12]. Furthermore, importance of the positive feedback for proper cell cycle regulation has also been proven in frog egg extracts [13]. Additional results in other organisms underlined the important role of the two positive feedback loops in the G2/M cell cycle transition [10], [14]–[16]. Mathematical and computational modeling further facilitated cell cycle research [17]–[19] and theoretical investigations of the feedback loops concluded that the joint effect of the two positive feedback loops can make the transitions even more robust [20]. Furthermore, it has been shown that the effects of the two loops (pure positive and double negative) are not totally equivalent [21], [22].

Already in 1990, Paul Nurse proposed that the control of G2/M transition is universal among eukaryotes [9]. Recent results support this idea [10], [15], [16] and extend it to the other cell cycle transitions [5], [6]. Indeed, further studies found that the G1/S transition is also controlled by positive feedback loop in budding yeast [23]–[25] and similar importance of positive feedbacks on the M/G1 transition were also discovered [26], [27]. Here we expand the universality concept and study a generic cell cycle transition regulatory system. Through computational modeling we investigate the dynamical differences between models with different transcriptional and post-translational control modes. Specifically, we analyze the transition dynamics in systems with periodic transcription of the activator or inhibitor, with single or double positive feedbacks and with cell cycle checkpoints acting on activators or inhibitors. We find that the effect of periodic transcriptional regulation on the activator or the inhibitor has the major impact on the dynamics.

## Results

**Figure 1:**
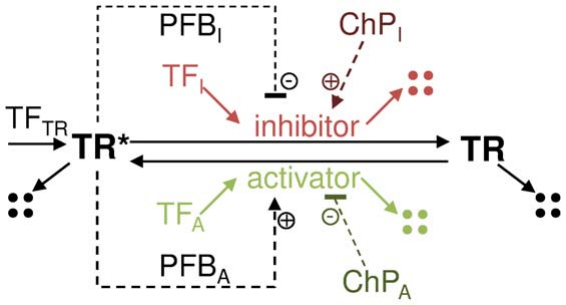
Regulation of a generic cell cycle transition regulator (TR) protein. TR, its activator and inhibitor all can be transcriptionally regulated (by TF_TR_, TF_A_ and TF_I_ respectively) as well as both the activator and inhibitor can be controlled by checkpoints (ChP_A_ and ChP_I_ respectively). Active form of the transition regulator (TR*) might activate its activator and/or inhibit its inhibitor, forming two positive feedback loops (PFB_A_ and PFB_I_). (Note that inhibiting an inhibitor is a positive effect leading to a double-negative = positive feedback loop). Solid lines represent reactions, dashed lines show regulatory effects. Positive feedbacks work on the post-translational level and catalyzed reactions have a non-catalyzed background rate, details for each individual reaction can be found in File S1.

Paul Nurse proposed that the control mechanism of G2/M transition is universal [9], here we investigate if the same picture holds true for all cell cycle transition regulatory modules. The unified cell cycle transition control system consists of an activator and an inhibitor, which control the activity of a transition regulator protein (TR on Fig. 1). The active form of the transition regulator (TR*) can activate its activator and/or inhibit its inhibitor – closing one or two positive feedback loops (PFB). All three components of this network could be transcriptionally regulated during the cell cycle, by various transcription factors (TFs on Fig. 1). A third layer of control on the system could come from checkpoints of the cell cycle (ChP), which ensure that a transition occurs only after an earlier cell cycle event has properly finished [1], [28]. These checkpoint signals stop the cell cycle transitions either by inhibiting the activator or activating the inhibitor [29], thus making it harder for the active transition regulator to turn on its positive feedback loops (Fig. 1). This wiring diagram consists of all possible transcriptional and post-translational regulatory interactions proposed for the cell cycle transition modules. Thus, Figure 1 presents all the well understood regulatory mechanisms that affect the dynamics of cell cycle transitions. For the detailed molecular mechanism of the proposed activation-inhibition steps, consult File S1.

### Literature data on regulation of cell cycle transitions

The universal G2/M control proposed by Nurse [9], fits this picture with Cdk/cyclins as transition regulators and Cdc25-Wee1 as the activator-inhibitor pair. Similar models have been proposed for the regulation of G1/S and M/G1 transitions, with the common pattern of the existence of one or more positive feedback loops [6]. Another common feature between transitions is that the activator-inhibitor pair often acts post-translationally, controlling the phosphorylation state of the transition regulator. In Table 1, we collected cell cycle transition regulators and their activators and inhibitors that are wired – fully or partially – in the generic way, presented in figure 1. Note that we do not investigate slower time scale regulations where a transition regulator is controlled by an activator or inhibitor which acts on its synthesis or degradation rate. We rather focus on cell cycle transitions where positive feedback works on the post-translational level. As table 1 shows, in fission and budding yeast and in humans all three cell cycle transitions have post-translational positive feedback loop control. Other crucial cell cycle events are also regulated by positive feedback loops [30], [31], but here we focus only on the mentioned three major cell cycle transitions.

**Table 1:** Cell cycle transition regulation in various organisms.

Transition	Organism	TR	Inhibitor	Activator	ChP	PFB
**G2/M**	**Fission yeast**	Cdc2/**Cdc13**	Wee1	**Cdc25**	**B**	**B**
	**Budding yeast**	Cdc28/**Clb2**	**Swe1**	Mih1	**I**	**B**
	**Fly**	Cdk1/CyclinB	Wee1, Myt1	**String**	**B**	**I**
	**Frog**	Cdc2/CyclinB	Wee1, Myt1	Cdc25	**B**	**B**
	**Human**	**Cdc2**/**CcnB1,2**	Wee1hu**Myt1**	hCdc25c	**B**	**B**
**M/G1**	**Budding yeast**	Cdh1, **Sic1**	Cdc28/**Clb2**	Cdc14	**A**	**I**
		***Pds1*** *^Inh^*	*Cdc14^#^*	*Cdc28/* ***Clb2^#^***	***I***	***I***
	**Fission yeast**	Wee1, (**Cdc25** inactivation)	Cdc2/**Cdc13**	Clp1	**A**	**I**
	**Human**	Wee1hu, (hCdc25c inactivation)	**Cdc2**/**CcnB1,2**	Cdc14A or PP2A	**A**	**B**
		Cdh1	**Cdc2**/**CcnB1,2**	Cdc14A	**A**	**I**
**G1/S**	**Budding yeast**	***Whi5*** *^Inh^*	*Cdc28/* ***Cln1,2,3***	*Cdc14*	***I***	***I***
	**Fission yeast**	Cdc2/**Cig2**	**Mik1**	Pyp3	**I**	**A**
	**Human**	Cdk2/Cyc**E**,A	Wee1hu	**hCdc25a**	**A**	**A**
		*Rb1^Inh^*	*Cdk6/CycD Cdk2/* ***CycE***	*PP1*	***I***	***I***

Cell cycle transition regulatory modules that resemble (in part or whole) the structure of Figure 1 were collected, together with the known information about periodic transcription, the existence of checkpoint and positive feedback regulation. Checkpoint regulation (ChP) and positive feedback loop (PFB) notation: **A**- acting through activator, **I** - through inhibitor, **B**- through both of them. **Bold** letters note genes that are periodically expressed during the cell cycle [2]. Note that all regulations are by phosphorylation - dephosphorylation reactions, with activators being phosphatases and inhibitors being kinases, except two reverse systems, noted by ^#^. ^Inh^ superscript and italic letters for the whole row means the TR is an inhibitor of the cell cycle transition, thus all effects on it are acting with reverse sign to the transition, furthermore an inhibitor of such a transition inhibitor is an indirect activator of the transition. (Detailed discussion and references for all of these findings can be found in File S1).

Our literature survey of Table 1 shows that two positive feedback loops were discovered in most organisms for G2/M transition regulations, but for some other transitions we find evidence for the existence of only one feedback loop. In these cases, we do not see a clear preference for positive feedback either through the activator or the inhibitor. Similar observations can be made on the effects of checkpoints on transitions: the most investigated G2/M transition has evidence for checkpoint signals affecting both inhibitors and activators, while in many other cases only one of the controllers is regulated by checkpoint signals – again without a clear preference towards activators or inhibitors. Based on theoretical analysis [20], one would think that the safest way to regulate cell cycle transitions is to use two feedback loops and have checkpoints which affect both regulators. Below we investigate if the lack of experimental evidence for the existence of an arrow on Figure 1 could have any biological importance.

It is important to notice in Table 1 that in all cases only one of the controllers (inhibitor or activator) of TR is expressed periodically during the cell cycle (noted with bold letters in Table 1). Again, we do not see a preference of transcriptional regulation of the activator or inhibitor in a database of high-throughput studies in numerous organisms [2]. The lack of evidence for a regulatory effect is not equal to evidence of the lack of such regulation; we might have incomplete knowledge of the systems, but it may also be that such variation in regulation is real and leads to biologically important dynamical differences.

### Comparing regulatory modes by computational modeling

To reveal if variation in the regulation can cause difference in the dynamics of cell-cycle transitions, we created a computational model of the generic network shown in Figure 1. We investigate *in silico* how the dynamic properties of the system are changing if one of the feedback loops is removed, how checkpoints can delay transitions and how the transcriptional control of the activator and inhibitor influences the dynamics. Furthermore, we test how reliably these transitions together with a negative feedback loop can give periodic oscillations – as expected from a robust cell cycle control system [13], [18].

We converted the regulatory network of Figure 1 into a computational model, using the BlenX programming language, which provides a framework that combines modular modeling and stochastic simulation capabilities [32]. Specifically, we created 24 models representing all combinations of: positive feedback on activator, inhibitor or both; transcription factor on activator or inhibitor; and checkpoint not induced, acting on activator or on inhibitor or on both. We assumed nonlinear enzymatic interactions (as do others [33]) between inhibitor/activator and their substrates. Although, the dynamics of the system would not change even if we were to use multisite phosphorylation to enhance nonlinearity of the feedback loops [21], [22].

### Two transcriptional control modes of cell cycle transitions

**Figure 2:**
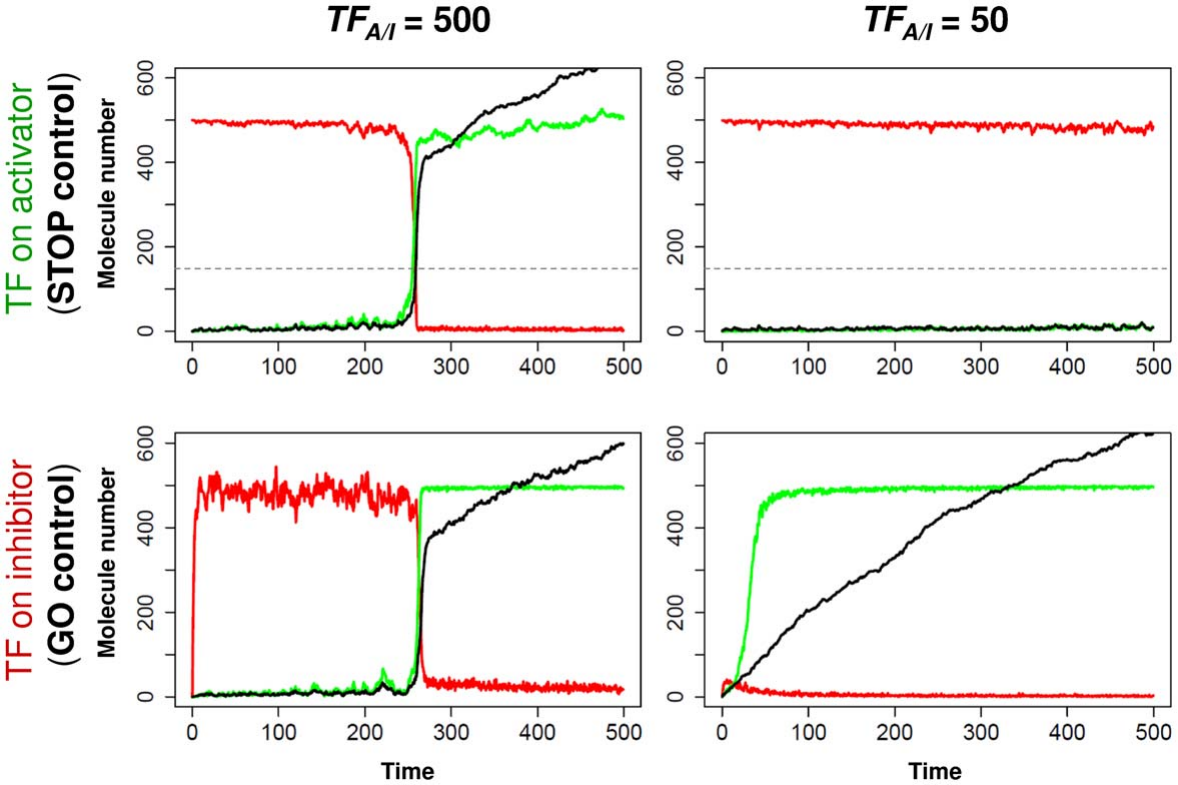
Transcriptional control modes of cell cycle transitions. Computational simulations of the system presented in figure 1 with transcription factor (TF) acting on the activator (upper panels) or on the inhibitor (lower panels) of TR, while the other regulator is assumed to be present in a constant total amount. At time = 0 we turned on the transcription of TR and of the activator or inhibitor with a highly active (left column) or a reduced (10%) activity (right column) of TF_A_ or TF_I_. Plotted are the molecule numbers of the active forms of: activator - green, inhibitor - red, TR* - black. At high TF level the two system behave similarly hitting the presumed TR* threshold (grey dashed line) at the same time, but at reduced transcriptional level they show totally different behavior. (Both positive feedbacks were working during these simulations, removal of one of them does not change the qualitative picture – see File S1). One can notice the elevated noise the transcriptional regulation causes in the activator and inhibitor levels.

The major finding as shown in Table 1 is that periodic transcription affects only one of the regulators. We do not see a general trend in which one of them is controlled transcriptionally. If a periodically induced inhibitor fails to be transcribed, but the activator is constantly present, the cell can proceed through the transition without a delay (Fig. 2 lower panels). Transcriptional control of the inhibitor is needed to stop/delay the transition and the default (periodic transcription independent) state of the system is to GO through the transition. This is what we see for the budding yeast G2/M, fission yeast G1/S and for various M/G1 transitions (see table 1 – note that for inhibitors of transitions (italic) the meaning should be reversed, since a GO for a transition inhibitor means STOP for the transition). These transitions are examples that cannot be fully stopped by a cell cycle checkpoint, eventually the cells “adapt” and proceed through the transitions, even though the checkpoint signal is still active [34]–[36]. In the simulations, we see that TR can be activated without a delay if the inhibitor is present in a low amount, as is in this case where the TR turns on its positive feedback loop(s) and keeps the inhibitor in its inactive form (Fig. 2)

If the activator is periodically expressed and the inhibitor is static, a failure in the periodic transcriptional program will inhibit the transition and without a high transcription of the activator it never happens (Fig. 2 upper panels). In this case, the positive feedback loop(s) of TR cannot fire, since the inhibitor is fully active. Without any activator, the TR cannot overcome this inhibition. Thus, the default message is to STOP the cell cycle if the periodic transcription is perturbed. Examples for this type of regulation include the G2/M control of fission yeast and the G1/S control of budding yeast cells (Table 1) in which transitions are blocked when the activators are missing [37], [38]. Note that in the case of the budding yeast G1/S control Whi5 is a TR that inhibits the transition and its inhibitor is periodically expressed, which leads to the STOP transcriptional control of the transition.

The above findings suggest that the most important transitions of the cell cycle are regulated by STOP transcriptional control of an activator that can be easily delayed in case of failure. In human cell cycle regulation, we explored the controls of the various forms of Cdc25: direct experiments showed that the level of the mitotic Cdc25c is constant, whereas the other forms are periodic [39]. In the view of the proposed GO and STOP regulations, this would suggest that human G1/S is the major control point with a STOP control and G2/M is less important with a GO control. The regulation of the restriction point transition inhibitor Rb1 also supports the idea that in human cells the G1/S transition is more carefully controlled by transcriptional regulation than the G2/M or M/G1 transitions.

The M/G1 transition is best characterized in budding yeast. The activation of Cdc20 induces a cascade of events that lead to Cdc14 activation [40], [41], which serves as the major activator of the irreversible exit of mitosis. The role of positive feedbacks in Sic1, Cdh1 and Pds1 regulation were established in recent years [26], [42], [43] and the importance of some of these proteins in the irreversibility of the transition was also proved [27]. Cdc14 inhibits the transition inhibitor Pds1 and activates the transition activators Sic1 and Cdh1 and periodically appearing Cdc28/Clb2 acts as an inhibitor of the transition – leading to a GO transcriptional control. Cdc28/Clb2 also affects Cdc14 activity directly [44], the introduction of such crosstalk do not influence our simulation results (not shown), still such feed-forward regulation could help the irreversibility of the transition [45], [46].

**Figure 3:**
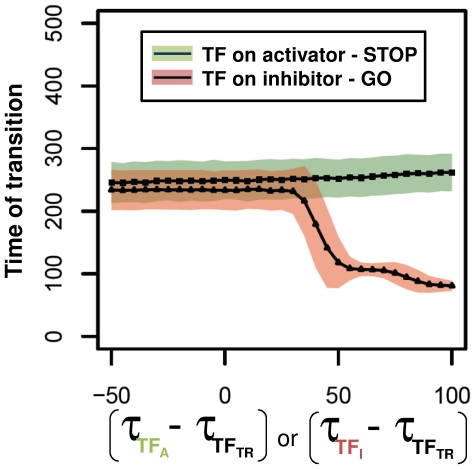
Effects of advance or delay in timing of transcriptional induction of activator or inhibitor. Time for the active form (TR*) to reach a threshold is registered versus the time difference between transcriptional initiation of the activator (green) or inhibitor (red). Rectangles show averages, shaded backgrounds show ± standard deviations from 1000 simulations at a given transcriptional advance (negative values on x-axis) or delay (positive values) compared to TR transcription.

As we found that most TRs are also periodically expressed during the cell cycle (table 1), we wanted to test how problems in transcriptional waves might influence the systems with the proposed two transcriptional regulatory modes. Stochastic simulations were initiated from the time point when TR transcription started, and we tested how the timing of the cell cycle transition (time for TR* to hit a critical value) depends on the time when the periodic regulator (activator or inhibitor) transcription is initiated. A delay (positive values on x-scale of Fig. 3) or advance (negative values) in the transcription of the activator compared to transcription of TR, causes less divergence. On the other hand, a bit of a delay in the inhibitor transcriptional induction (GO control) can cause a large advance in the timing of cell cycle transitions (Fig. 3). This difference between the two systems is the result of positive feedback loops which lock the transition controllers in either one of two stable states. In one state, the inhibitor is active, TR is inactive and the activator is inactive. In the other state, TR can turn its loop with the active activator ON causing the inactivation of the inhibitor. In which of the two steady states the system locks depend on the initial state and on the activator and inhibitor levels.

**Figure 4:**
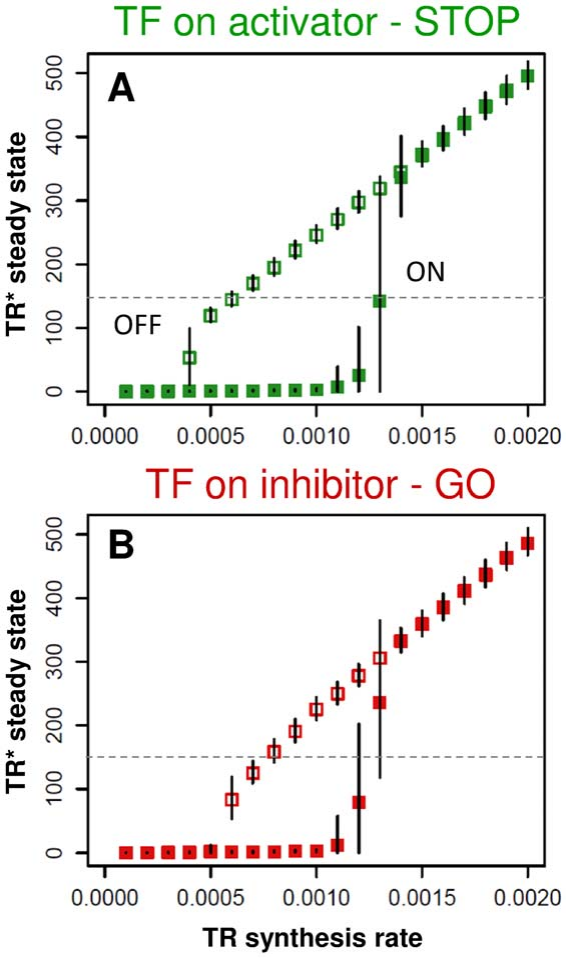
Bistability in cell cycle transitions under various transcriptional control modes. Similarly to experimental investigations of bistability of cell cycle transitions [11], [12], here we plot the *in silico* calculated average steady state molecular levels of the active form TR* when its synthesis rate was moved from lower to higher values (filled rectangles) or when it was moved from high to low values (empty rectangles). Error bars show ± standard deviation of 100 simulations at each input values. (**A**) TF_A_ is active and inhibitor level is constant (STOP control), (**B**) the other way around (GO control). Grey dashed lines show an idealized threshold value, above this level TR* induces the cell cycle transition. When TR synthesis is increasing both models show a sharp ON transition when TR synthesis crosses ∼0.0013 (we set the flexible parameters of the models to get this value approximately equal in all cases).

To better see the significance of the positive feedback loops, we characterize the bistability of cell cycle transitions [11], [12], [24] in the various models with different regulations. Figure 4 shows that the transcriptional STOP and GO controls do not show great differences in bistability - measured by the averages (± standard deviation) of stochastic simulations with slowly increasing or decreasing TR synthesis rate [47]. A small reduction in the bistable regime (thus the robustness of the switch) for GO controlled model however could be observed. Still, we conclude that transcriptional regulation has a minor role in the bistability of cell cycle transitions. Plots shown in figure 4 were created from both positive feedback loops present in the system. In File S1, we show that one positive feedback is enough to create bistability and the bistable regions are quite similar in GO and STOP controlled systems. Still with one positive feedback the bistability is reduced compared to the two loops system [20].

**Figure 5:**
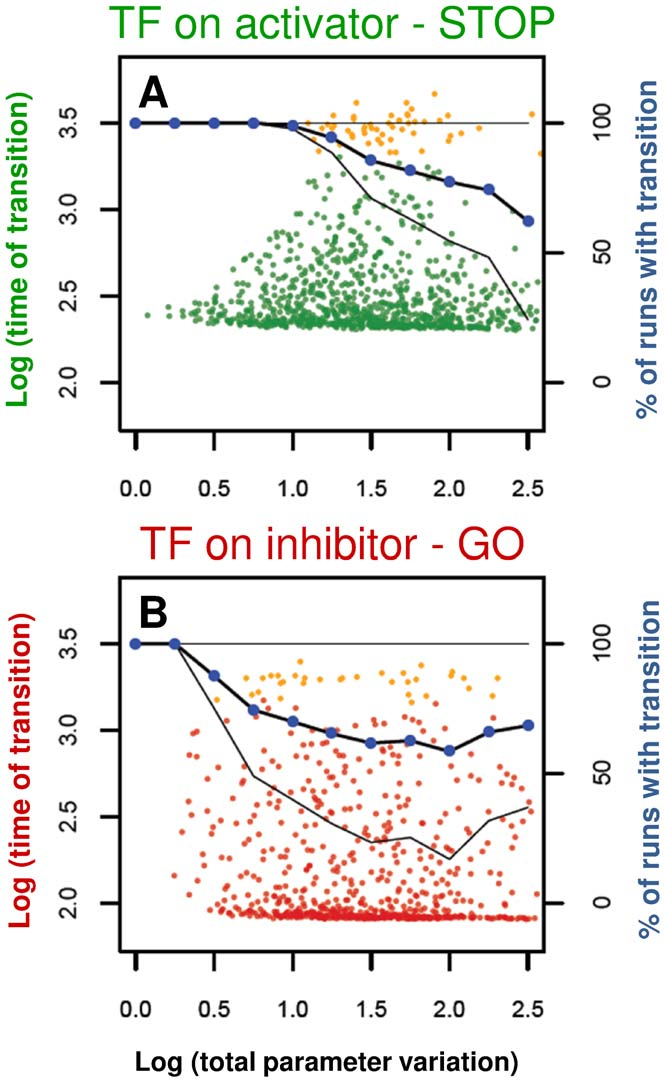
Parameter robustness test of the models. We tested how extrinsic parameter variations in the regulation of the transcriptionally controlled proteins influence the timing of cell cycle transitions. The parameters that control synthesis and degradation of the activator (**A**) or inhibitor (**B**) were randomly sampled (1000 parameter sets) between one tenth and ten times the basal values and the variations in the timing of the transitions are reported versus a measure of parameter variation distance as earlier defined [68]. Each colored dot represents the average of 100 parallel stochastic simulations at a randomly drawn parameter set, orange dots stand for parameter combinations where not all 100 simulations gave successful transitions (TR* hitting the critical value). Connected blue dots give the average percentage of successful transitions, with black lines giving ± standard deviation (corresponding values on the right y-axis).

Since our model uses arbitrary parameter values that were selected in order to get a sharp threshold for TR activation (at the same TR synthesis rate – see Fig. 4), we were interested in how robustly these sharp cell cycle transitions are preserved for parameter variations. We find (Fig. 5) that similarly to the results presented above, the model with transcriptional regulation of the activator (STOP control) leads to lower noise for parameter variations compared to systems with transcriptional regulation of the inhibitor (GO control). We see this trend both in the increased spread on the timing of successful transitions and in the decreased percentage of successful transitions as parameter variation increases (dots and solid line respectively on Fig. 5). As the bistability test also suggested above, the presence of both positive feedback loops give a model with the best parameter robustness, but its advantage compared to a single positive feedback system is minimal (File S1). Thus, we conclude that robustness of cell cycle transitions depend most on the modes of transcriptional control as long as at least one strong positive feedback is present in the system.

Next, we test how reliably the various model versions provide a cell cycle transition that can support robust cell cycle oscillations. We connected the cell cycle transition models to a minimal negative feedback loop model [48], where a high level of TR* induces its own degradation. Such combination of positive and negative feedback loops is expected to give a robust minimal cell cycle oscillator [13], [18], [49]. We observe that in the presence of both positive feedback loops, the two transcriptional regulations do not show relevant differences in oscillation robustness, but the combination of transcriptional regulation and positive feedback both acting on the inhibitor cannot provide reliable oscillations (File S1). Thus, we conclude that in the case of absence of positive feedback on the activator, the STOP controlled (TF on activator) cell cycle transitions more reliably provide a robust control in oscillating cell cycles.

**Figure 6:**
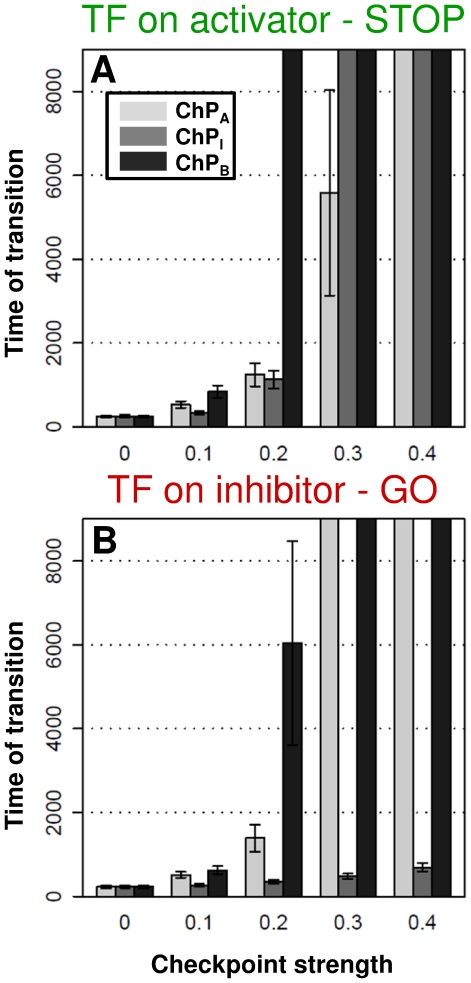
Checkpoint efficiency on various versions of cell cycle transition control models. ChP_A_ of figure 1 is inhibiting the activator of the TR, while ChP_I_ moves the inhibitor into a form that is more active in inhibiting TR* [69] and ChP_B_ labels results when both checkpoints are effective with similar strength (see File S1 for more details). We plot the average times of cell cycle transitions (and with error bars the ± standard deviation) of 1000 stochastic simulations for each model version. Where the columns exceed the plot height, transitions did not occur in >90% of the simulations, so here the checkpoints hold tightly.

As Figure 1 and Table 1 show, checkpoints of the cell cycle can act either by up-regulating the inhibitors or down-regulating the activators or both. We computationally check how the three types of checkpoint signaling can delay the transitions in the various versions of the model. In Figure 6, we plot how long different strength checkpoints can delay cell cycle transitions. In most cases, the STOP control gives a tighter checkpoint block than a GO control, especially in the case when the checkpoint acts only on the inhibitor. Even a strong checkpoint signal on the inhibitor is unable to block the transition in a GO control model (Fig. 6B), while in a STOP control model the same checkpoint strength could be enough to block the transition indefinitely (Fig. 6A). We conclude that systems with checkpoints acting only on the inhibitor and transcriptional control also affecting the inhibitor, cannot give a reliable cell cycle block. This is the case for the budding yeast G2/M control system (Table 1), which can adapt and leak through the morphogenesis checkpoint [36]. If only one of the positive feedbacks is present then the trends are similar: transcription and checkpoint both on inhibitor are ineffective in stopping the transition (File S1), thus major differences by the loss of one feedback cannot be noticed. We conclude that in the case of transcriptional regulation on the inhibitor, the checkpoint should act on the activator or on both regulators in order to give a solid cell cycle block. Cell cycle transitions with transcriptional control of the activator can be better stopped by the checkpoint acting either on the activator or inhibitor.

## Discussion

The key regulatory components of the cell cycle were discovered more than 30 years ago [50] and the universal picture that positive feedback loops regulate mitotic entry has gradually emerged [9], [18], [19], [51]. Here we investigated how far this universality holds for all cell cycle transitions in some of the most well studied organisms. Our computational modeling results suggest that there are crucial differences in transition dynamics if periodic transcription acts on the activator or inhibitor of the transition. The exact details of checkpoint and positive feedback regulation are not that crucial for proper cell cycle transitions, still co-existence of the two feedback loops makes the transitions more robust and checkpoints acting on both regulators are more capable of stopping the transitions. Our literature survey shows that there is no evidence for the existence for such double regulations in all investigated organisms at various cell cycle transitions.

The major differences between cell cycle transitions are in the transcriptional regulation of the activator and inhibitor of the transition regulators. In all investigated cases only one is regulated periodically during the cell cycle (Table 1). The computational analysis shows that the transcriptional regulation of the inhibitor leads to a systems that is less robust for transcriptional delays or parameter variations and less responsive for checkpoint controls; furthermore, it is less effective to serve as the regulator of a single transition in a cell cycle oscillator. Thus, we termed this as “GO control”, as it is effective in passing through the transition even in the case of a failure. By contrast, “STOP control” is achieved by transcriptional regulation of the activator. This module does not allow the transition to happen in case of a failure and gives a higher robustness of the transition in all investigated tests. Thus, our computational analysis predicts that the most important cell cycle transitions need to be regulated by STOP control. Indeed the G2/M control of fission yeast cells and G1/S control of budding yeast and human cells are under STOP control (Table 1 - also note that a GO control of a transition inhibitor is a STOP signal for the transition). These are the most crucial control points of the cell cycle of these organisms [1]. On the other hand, some cell cycle transitions are much less carefully controlled by a GO control as we see in some cases (Table 1). Various checkpoints in yeasts and higher eukaryotes can adapt and allow the cells to proceed even in the case of a failure and leave the repair for later times [34], [35]. Our analysis suggests that in these cases, a GO transcriptional control works together with a checkpoint working only on the inhibitor. Indeed in the budding yeast G2/M transition and morphogenesis checkpoint is controlled by a checkpoint that acts only on the inhibitor and has a GO transcriptional control [2], [36], [52].

On the other hand, the most reliable transitions we observe are when both positive feedbacks are working and when checkpoints act on both regulators. One would expect to see this setup for all of the important transitions and indeed for the most investigated G2/M transitions we found all the needed pieces of evidence [20], [21]. Maybe we just lack the key experiments from other organisms, but it also could be that evolution found these double regulations too expensive and solved it with a cheaper - although a bit less reliable - system. Our analysis suggests that the most reliable, although more economical solution is the use of the positive feedback through the inhibitor, the checkpoint on the activator together with a STOP transcriptional control on the activator. Some recent evidence supports these findings as the positive feedback loop through the inhibition of the inhibitor was suggested to be the most important for the robustness of the transitions [14], [22], [53], [54] and the activator, Cdc25 was suggested as the major target of the mitotic checkpoint [39], [55]. It is also worth noticing that in most cases phosphatases are the activators of TR, which itself is often a kinase, in particular a cyclin-dependent kinase. Importance of phosphatases for M/G1 transition has been already discussed [56], our analysis suggests that they might be generally important for cell cycle transitions.

We collected data in Table 1 from experiments that were indeed performed in the given cell type. During our literature review, we noticed that many papers use results from experiments on other organisms to build their further investigations on different cell types; e.g. considering the effect of frog PP2a on Cdk targets [57] as a starting point of investigations of human cells [58]. Such merging of experimental results from different organisms could lead to a universal picture, but until all experiments are performed on a given organism we cannot be sure if the lack of a link compared to the universal network of figure 1 is a consequence of lack of knowledge or a result of special dynamical or economical constraints.

Following the observation that we did not find a single case in which both regulators are periodically expressed, we further speculate that the periodic transcription of crucial regulators might have been a subject of selection. If either the activator or inhibitor is more often needed in the life cycle of the cell, then this protein might be selected for constant transcription, while proteins with lower demand might keep periodic transcriptional regulation [59]–[61]. Such thinking suggests that cell cycle transitions that are usually passed quickly are selected for GO transcriptional control while transitions that are halted for longer times are under STOP control. The two yeast systems perfectly fit this picture with budding yeast having GO control in G2/M and STOP at G1/S and fission yeast having it the opposite way, but having its critical transition at G2/M compared to budding yeast with an essential G1/S control.

Following our findings on lack of evidence to support a universal view of all cell cycle transitions, we propose to investigate more carefully if a cell cycle transition regulatory effect is conserved between organisms. We present a unified picture of all possible transcriptional and post-translational controls on cell cycle transition regulators (Fig. 1), but parts of this interaction network might be missing from some of the transition regulatory networks in various organisms. Depending on which part of the system is missing, it can have different effect on transition dynamics. This could be an explanation for the observed differences in the cell cycle regulation of different organism. Indeed, recent results in plants show that the regulatory network interactions greatly differ from the yeast or metazoan systems [62] and even in the yeast there are some opposing ideas about the importance of some of the interactions [63], [64]. Such uncertainty in the presence or absence of some regulations might cause a problem in understanding cell cycle regulation. For instance, variations in transcriptional regulation could have a major impact on differentiated mammalian cells, where different cell types in the same organism have different transcriptional profiles [65]. Our results suggest that such transcriptional alterations of cell cycle transition regulators can cause a major change in the dynamics of these transitions.

## Methods

In this section, we give a high-level explanation of the methods we used. A more detailed description can be found in File S1.

### Model development

We built models of cell cycle transition regulations representing different combinations of three regulatory effects such as transcription, post-translational positive feedback and checkpoint. Transcription factors can act on the activator or on the inhibitor (2 sub-model types); positive feedback can work through the activator, through the inhibitor or both (3 sub-model types) and checkpoints can be absent or act on activator or inhibitor or on both (4 sub-model types). All combinations of these lead to 24 models. In the main text, we mainly discuss the models where both positive feedbacks are active while the models with only one positive feedback are mainly discussed in File S1. Also in File S1, we discuss the extension of the basic 6 models (no checkpoints) by a negative feedback loop.

### Model implementation

All the models have been created using the BlenX programming language [32] and simulated by means of the Beta Workbench [66]. BlenX is a language based on process calculi and rule-based paradigms. It is a stochastic language in the sense that the probability and speed of the interactions are specified in the program. In this respect, we solve the models by a stochastic simulator based on an efficient variant of the Gillespie algorithm [67]. In File S1, we provide detailed description of the simulation methods of results presented in the figures 2, 3, 4, 5, 6.

## Supporting Information

File S1 **Supplementary text containing and extended version of** **Table 1** **with references, details on model development and implementation.** Here we also describe simulation methods and details on the main figures of the paper with 7 figures and 7 tables.
(PDF)
