# Supplementary material for: Transcriptional Regulation Is a Major Controller of Cell Cycle Transition Dynamics
Source: PLoS One. 2012 Jan 6;7(1):e29716. doi: 10.1371/journal.pone.0029716 (PMC3253096; doi:10.1371/journal.pone.0029716)
Supplement: File S1 — Supplementary text containing and extended version of Table 1 with references, details on model development and implementation. Here we also describe simulation methods and details on the main figures of the paper with 7 figures and 7 tables. (PDF) [file pone.0029716.s001.pdf]

Supplementary materials to the paper:

Transcriptional regulation is a major controller of cell cycle transition dynamics

Alessandro Romanel, Lars Juhl Jensen, Luca Cardelli, Attila Csikász-Nagy

Contents

1. Extended version of Table 1 2
2. Model development 4
3. Model implementation 8
4. Simulations of GO and STOP transcriptional regulations 10
5. Simulation methods and details on the main figures of the paper 11
6. Oscillations 15
7. Supplementary references 17

Including 7 supplementary figures and 7 supplementary tables.

1. Extended version of Table 1

Transition	Organism	TR	Inhibitor	Reference TR – I interaction	Activator	Reference TR – A interaction	ChP	Reference ChP	PFB	References PFB
G2/M	**Fission yeast** *S. pombe*	Cdc2/**Cdc13**	Wee1	(Aligue *et al*, 1997; Russell and Nurse, 1987)	**Cdc25**	(Kovelman and Russell, 1996; Russell and Nurse, 1986)	**B**	(O'Connell *et al*, 2000; Rhind and Russell, 1998)	**B**	(Aligue *et al*, 1997; Kovelman *et al*, 1996)
	**Budding yeast** *S. cerevisiae*	Cdc28/**Clb2**	**Swe1**	(Harvey *et al*, 2005)	Mih1	(Pal *et al*, 2008)	**I**	(Sia *et al*, 1998)	**B**	(Booher *et al*, 1993; Pal *et al*, 2008)
	**Fly** *D. melanogaster*	Cdk1/CyclinB	Wee1, Myt1	(Campbell *et al*, 1995; Stumpff *et al*, 2004)	**String**	(Edgar and O'Farrell, 1990)	**B**	(Sibon *et al*, 1997)	**I**	(Price *et al*, 2000)
	**Frog** *X. laevis*	Cdc2/CyclinB	Wee1, Myt1	(Mueller *et al*, 1995)	Cdc25	(Kumagai and Dunphy, 1992)	**B**	(Kumagai and Dunphy, 1999; Stanford and Ruderman, 2005)	**B**	(Coleman and Dunphy, 1994; Karaiskou *et al*, 1998)
	**Human** *H. sapiens*	**Cdc2/CcnB1,2**	Wee1hu **Myt1**	(Parker and Piwnica-Worms, 1992; Watanabe *et al*, 2005; Watanabe *et al*, 1995)	hCdc25c	(Hoffmann *et al*, 1993)	**B**	(Deibler and Kirschner, 2010; Donzelli and Draetta, 2003; Lukas *et al*, 2004; Sanchez *et al*, 1997)	**B**	(Deibler *et al*, 2010; Enders, 2010)
M/G1	**Budding yeast** *S. cerevisiae*	Cdh1, **Sic1**	Cdc28/**Clb2**	(Nash *et al*, 2001; Zachariae *et al*, 1998)	Cdc14	(Jaspersen *et al*, 1999; Visintin *et al*, 1998)	**A**	(Queralt and Uhlmann, 2008; Yoshida *et al*, 2002)	**I**	(Amon, 1997; Visintin *et al*, 1997)
		Pds1 *(INH)*	*Cdc14#*	*(Visintin et al, 1998)*	*Cdc28/**Clb2**#*	*(Agarwal and Cohen-Fix, 2002)*	***I***	*(Queralt et al, 2008; Yoshida et al, 2002)*	***I***	*(Holt et al, 2008)*
	**Fission yeast** *S. pombe*	Wee1, (**Cdc25** inactivation)	Cdc2/**Cdc13**	(Aligue *et al*, 1997; Kovelman *et al*, 1996)	Clp1	(Esteban *et al*, 2004; Wolfe and Gould, 2004)	**A**	(Chen *et al*, 2008; Mishra *et al*, 2004; Wolfe *et al*, 2006)	**I**	(Aligue *et al*, 1997; Kovelman *et al*, 1996)

Transition	Organism	TR	Inhibitor	Reference TR – I interaction	Activator	Reference TR – A interaction	ChP*	Reference ChP	PFB *	References PFB
	**Human** *H. sapiens*	Wee1hu (hCdc25c inactivation)	**Cdc2/ CcnB1,2**	(D'Angiolella *et al*, 2007; Potapova *et al*, 2009)	Cdc14A or PP2A	(Bollen *et al*, 2009; Burgess *et al*, 2010; Krasinska *et al*, 2007; Queralt *et al*, 2008)	A	(Mailand *et al*, 2002)	B	(Burgess *et al*, 2010; Kapuy *et al*, 2009; Potapova *et al*, 2009)
		Cdh1	**Cdc2/ CcnB1,2**	(Kramer *et al*, 2000)	Cdc14A	(Bembenek and Yu, 2001)	A	(Mailand *et al*, 2002)	I	(Kramer *et al*, 2000)
G1/S	**Budding yeast** *S. cerevisiae*	**Swi6**	Cdc14#	(Bloom and Cross, 2007; Geymonat *et al*, 2004)	Cdc28/**Clb6**#	(Schwob and Nasmyth, 1993)	A	(Palou *et al*, 2010)	A	(Schwob *et al*, 1993)
		Whi5 *(INH)*	*Cdc28/**Cln3***	*(Costanzo et al, 2004; de Bruin et al, 2004)*	*Cdc14*	*(Taberner et al, 2009)*	I	*(Tyers and Futcher, 1993)*	I	*(Costanzo et al, 2004; de Bruin et al, 2004)*
	**Fission yeast** *S. pombe*	Cdc2/**Cig2**	**Mik1**	(Zarzov *et al*, 2002)	Pyp3	(Millar *et al*, 1992)	I	(Murakami and Nurse, 2000)	A	(Sveiczer *et al*, 1999)
	**Human** *H. sapiens*	Cdk2/Cyc**E**,A	Wee1hu	(Parker *et al*, 1992; Watanabe *et al*, 2005; Watanabe *et al*, 1995)	**hCdc25a**	(Boutros *et al*, 2006)	A	(Donzelli *et al*, 2003; Lukas *et al*, 2004; Mailand *et al*, 2000)	A	(Hoffmann *et al*, 1994)
		Rb1 (INH)	*Cdk6/CycD Cdk2/**CycE***	*(Lundberg and Weinberg, 1998)*	*PP1*	*(Durfee et al, 1993)*	I	*(Wade Harper et al, 1993)*	I	*(Geng et al, 1996)*

Table S1. Cell cycle transition regulators in various organisms, including references for all claims. Similar to Table 1 of the main text, just here we add references that support our claims on the roles of activator and/or inhibitors in checkpoint regulation (ChP) and their positive feedback (PFB) interactions with the transition regulators (TR). Checkpoint regulation (ChP) and positive feedback loop (PFB) notation: **A**- acting through activator, **I**- through inhibitor, **B**- through both of them. Blue, bold letters note genes that are periodically expressed during the cell cycle in system wide studies (Gauthier *et al*, 2010); controversially to this hCdc25c was found static in individual experiments (Donzelli *et al*, 2003). Note that all regulations are by phosphorylation – dephosphorylation reactions, with activators being phosphatases and inhibitors being kinases, except two reverse systems, noted by #. At the M/G1 transition of fission yeast cells Wee1 is activated and Cdc25 is inactivated after dephosphorylation by Clp1 (Wolfe *et al*, 2004), similarly Wee1hu is activated and Cdc25c is inhibited during the human M/G1 transition (Potapova *et al*, 2009) these effects are lumped together in one row of this table. (INH) sign and italic letters for the whole row means the TR is an inhibitor of the cell cycle transition, thus all effects on it are acting with reverse sign to the transition.

2. Model development

Here we present in detail the various models we used in the paper. Figure S1 shows a complete picture of all the main species and all their interactions $R_i(0 < i < 29)$ the kinetics laws of which are detailed in Table S2.

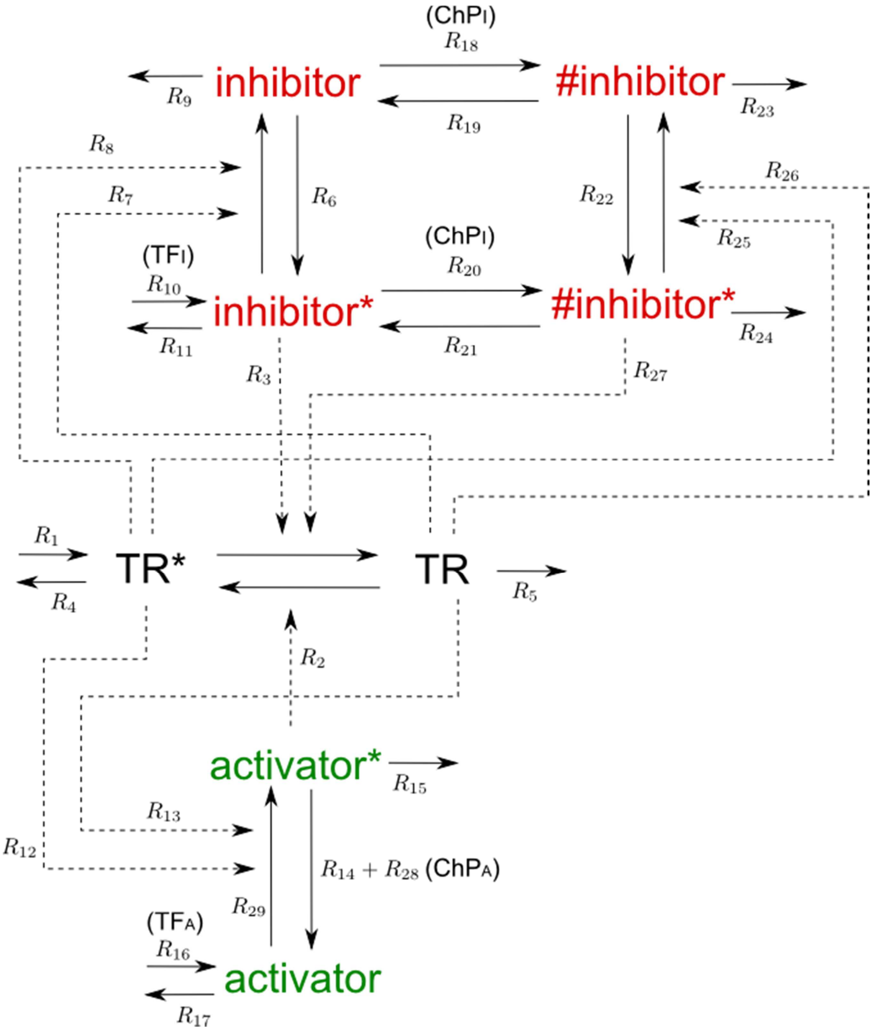

Figure S1: Detailed interaction map of the generic cell cycle transition regulator. *Solid arrows represent reactions, while dashed arrows represent activating effects. In brackets we note the reactions where transcription factors (TFs) and checkpoint proteins (ChPs) act.*

In the model notation we follow, *TR** and *TR* represent, respectively, the active and inactive forms of the transition regulator, *inhibitor** and *inhibitor* represent, respectively, the active and inactive forms of the inhibitor and *activator** and *activator* represent, respectively, the active and inactive forms of the activator. In detail, *TR** activates *activator* and inhibits *inhibitor**. Although *TR* (the inactive form of the transition protein) acts on the activator and the inhibitor in the same way as *TR**, its efficiency is 100 times lower, as proposed by others after theoretical and experimental observations (Ciliberto *et al*, 2007; Deibler *et al*, 2010). Moreover, note that both the activity of *TR** (and *TR*) on *inhibitor** and on *activator* form positive feedbacks (PFB); *activator** activates *TR** and *inhibitor* inhibits it. The checkpoint moves inhibitor to a hyperactive form, *#inhibitor* that is four times stronger inhibitor of *TR** than the normally active (but not checkpoint affected) *inhibitor** form. This way of implementing checkpoint activation was taken from an earlier model of morphogenetic checkpoint of budding yeast cells (Ciliberto *et al*, 2003).

$R_1 = \frac{kms}{\alpha}$	$R_{16} = kcs \times TF$
$R_2 = \frac{kma \times \alpha \times activator^* \times TR}{jma + (\alpha \times TR)}$	$R_{17} = kcd_1 \times activator$
$R_3 = \frac{kmi \times \alpha \times inhibitor^* \times TR^*}{jmi + (\alpha \times TR^*)}$	$R_{18} = \frac{kcp_7 \times \alpha \times ChPI \times inhibitor}{jcp_7 + (\alpha \times inhibitor)}$
$R_4 = kmd \times TR^*$	$R_{19} = \frac{kcp_6 \times \alpha \times Pho \times \#inhibitor}{jcp_6 + (\alpha \times \#inhibitor)}$
$R_5 = kmd_1 \times TR$	$R_{20} = \frac{kcp_1 \times \alpha \times ChPI \times inhibitor^*}{jcp_1 + (\alpha \times inhibitor^*)}$
$R_6 = \frac{kwa \times \alpha \times E_1 \times inhibitor}{jwa + (\alpha \times inhibitor)}$	$R_{21} = \frac{kcp_2 \times \alpha \times Pho \times \#inhibitor^*}{jcp_2 + (\alpha \times \#inhibitor^*)}$
$R_7 = \frac{kwi \times \alpha \times TR^* \times inhibitor^*}{jwi + (\alpha \times inhibitor^*)}$	$R_{22} = \frac{kcp_5 \times \alpha \times E_1 \times \#inhibitor}{jcp_5 + (\alpha \times \#inhibitor)}$
$R_8 = \frac{kwi \times \alpha \times perc \times TR \times inhibitor^*}{jwi + (\alpha \times inhibitor^*)}$	$R_{23} = kwd_1 \times \#inhibitor$
$R_9 = kwd_1 \times inhibitor$	$R_{24} = kwd \times \#inhibitor^*$
$R_{10} = kws \times TF$	$R_{25} = \frac{kcp_3 \times \alpha \times TR^* \times \#inhibitor^*}{jcp_3 + (\alpha \times \#inhibitor^*)}$
$R_{11} = kwd \times inhibitor^*$	$R_{26} = \frac{kcp_4 \times \alpha \times perc \times TR \times \#inhibitor^*}{jcp_4 + (\alpha \times \#inhibitor^*)}$
$R_{12} = \frac{kca \times \alpha \times TR^* \times activator}{jca + (\alpha \times activator)}$	$R_{27} = \frac{kmi_1 \times \alpha \times \#inhibitor^* \times TR^*}{jmi_1 + (\alpha \times TR^*)}$
$R_{13} = \frac{kca \times \alpha \times perc \times TR \times activator}{jca + (\alpha \times activator)}$	$R_{28} = \frac{kcp_8 \times \alpha \times ChPA \times activator^*}{jcp_8 + (\alpha \times activator^*)}$
$R_{14} = \frac{kci \times \alpha \times E_2 \times activator^*}{jci + (\alpha \times activator^*)}$	$R_{29} = \frac{kca \times \alpha \times S \times activator}{jca + (\alpha \times activator)}$
$R_{15} = kcd \times activator^*$	

Table S2: Kinetic laws associated to reactions, corresponding to notation on Figure S1. *The values associated to basal parameters are reported in Table S3.*

Syntheses and degradations follow the law of mass action, while all the other reactions follow the Michaelis-Menten kinetics; parameters starting with character *k* are the catalytic constants (dimension 1/min), while parameters starting with *j* are the Michaelis constants (dimensionless). Moreover, since we describe systems in terms of explicit molecule counts and not in terms of concentrations, we introduce a scaling factor α which is used to represent

the size of the system - volume of the cell, as described earlier (Mura and Csikasz-Nagy, 2008).

$kms = 0.004$	$kca = 2$	$kcd = 0.01$	$kcp_1 = 0.1$
$kma = 0.5$	$jca = 0.1$	$kcd_1 = 0.01$	$kcp_2 = 0.2$
$jma = 1$	$kci = 0.2$	$jcp_1 = 2$	$kcp_3 = 2$
$kmi = 0.5$	$jci = 0.1$	$jcp_2 = 0.1$	$kcp_4 = 2$
$jmi = 1$	$kmi_1 = 2$	$jcp_3 = 0.1$	$kcp_5 = 0.2$
$kmd = 0.002$	$kws = 0.5$	$jcp_4 = 0.1$	$kcp_6 = 0.2$
$kmd_1 = 0.002$	$kwd = 0.5$	$jcp_5 = 0.1$	$kcp_7 = 0.2$
$kwa = 0.2$	$kwd_1 = 2$	$jcp_6 = 0.1$	$kcp_8 = 0.1$
$jwa = 0.1$	$jwi = 0.1$	$jcp_7 = 0.1$	$perc = 0.01$
$kwi = 2$	$kcs = 0.01$	$jcp_8 = 0.1$	$\alpha = 1/500$

Table S3: Basal parameters set.

All the molecular species that are present in the expressions of Tables S2 but not in Figure S1 have constant population, representing background activities of constantly present kinases and phosphatases. In particular, *E1* activates the *inhibitor* (and also *#inhibitor*), while *E2* inhibits the *activator**, both are needed to give a threshold for the autocatalytic TR* induced autocatalysis. Moreover, *S* is representing a background signal, activating *activator* in case the PFB_A is removed and there is no activation of *activator* by *TR**. Species ChP_A and ChP_I are introduced to represent checkpoints on the activator and the inhibitor, respectively. While ChP_A promotes the inhibition of the *activator*, ChP_I transforms the *inhibitor* into the more active *#inhibitor* form (see above).

When we say that TF_I acts on the *inhibitor* we introduce the synthesis reaction R_{10} and all the corresponding degradation reactions R_9, R_{11}, R_{23} and R_{24}. In the same way, when we say that TF_A acts on the activator we refer to the synthesis reaction R_{16} and all the corresponding degradation reactions R_{15} and R_{17}. In case there is no transcriptional regulation of the *activator* or *inhibitor* then we keep the above parameters at 0 and use a constant high (500 molecules) total activator or inhibitor level (see Table S4 for details).

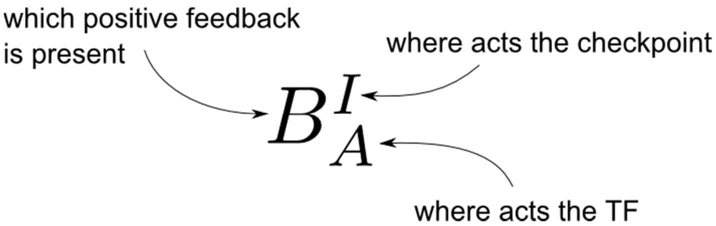

Explanation of model notation. *The possible 24 combination of models are represented by the following way: the main character tells which positive feedback is present in the model (e.g. in the example B means that both positive feedbacks are present); the subscript character tells weather TF acts on the inhibitor or on the activator (e.g. in the example on the activator); the superscript character tells where acts the checkpoint (e.g. in the example the checkpoint acts on the inhibitor).*

Model	*inhibitor**	*activator*	TF_{TR}	*E1*	*E2*	ChP_A	ChP_I	*Pho*	*S*
B_A	500	0	500	500	500	0	0	0	0

B_I	0	500	500	500	500	0	0	0	0
B_A^A	500	0	500	500	500	500	0	0	0
B_I^A	0	500	500	500	500	500	0	0	0
B_A^I	500	0	500	500	500	0	500	500	0
B_I^I	0	500	500	500	500	0	500	500	0
B_A^B	500	0	500	500	500	500	500	500	0
B_I^B	0	500	500	500	500	500	500	500	0
I_A	500	0	500	500	500	0	0	0	30
I_I	0	500	500	500	500	0	0	0	40
I_A^A	500	0	500	500	500	500	0	0	30
I_I^A	0	500	500	500	500	500	0	0	40
I_A^I	500	0	500	500	500	0	500	500	30
I_I^I	0	500	500	500	500	0	500	500	40
I_A^B	500	0	500	500	500	500	500	500	30
I_I^B	0	500	500	500	500	500	500	500	40
A_A	500	0	500	500	500	0	0	0	0
A_I	0	500	500	500	500	0	0	0	0
A_A^A	500	0	500	500	500	500	0	0	0
A_I^A	0	500	500	500	500	500	0	0	0
A_A^I	500	0	500	500	500	0	500	500	0
A_I^I	0	500	500	500	500	0	500	500	0
A_A^B	500	0	500	500	500	500	500	500	0
A_I^B	0	500	500	500	500	500	500	500	0

Table S4: Initial conditions (in molecule numbers) for all the possible model combinations. *The initial populations of molecular species not present in the table is always 0. This means that we assume that before the transition the activator is either not present (but actively transcribed), or present in its inactive form. The inhibitor is either not present (but actively transcribed) or present in its active form. Meaning that not periodically transcribed activator and inhibitor molecules are assumed to be present in TR unaffected form in high level (500 molecules). Details on the model notations are given above.*

The 24 different models represent combinations of the three regulatory effects (transcription, positive feedback and checkpoint):

- we considered two models, denoted by B_A and B_I , that have PFB both on the inhibitor and the activator, have no checkpoints (i.e., ChP_A and ChP_I set to 0) and are such that one (B_A) has TF acting only on the activator and the other (B_I) has *TF* acting only on the inhibitor. Then we consider models pairs B_A^A, B_I^A and B_A^I, B_I^I that are obtained from B_A and B_I by adding checkpoint ChP_A or checkpoint ChP_I, respectively;

- we considered two models, denoted by I_A and I_I, that have PFB only on the inhibitor, have no checkpoints (i.e., ChP_A and ChP_I set to 0) and are such that one (I_A) has *TF* acting only on the activator and the other (I_I) has *TF* acting only on the inhibitor.

Then we consider models pairs I_A^A, I_I^A and I_A^I, I_I^I and I_A^B, I_I^B that are obtained from I_A and I_I by adding, respectively, checkpoint ChP_A, checkpoint ChP_I or both the checkpoints – here we needed to introduce molecule *S* to induce some activation of *activator* in the absence of PFB on *activator*;

- we considered two models, denoted by A_A and A_I, that have PFB only on the activator, have no checkpoints (i.e., ChP_A and ChP_I set to 0) and are such that one (A_A) has *TF* acting only on the activator and the other (A_I) has *TF* acting only on the inhibitor. Then we consider models pairs A_A^A, A_I^A and A_A^I, A_I^I that are obtained from A_A and A_I by adding checkpoint ChP_A or checkpoint ChP_I, respectively.

3. Model implementation

All the models presented in the previous section have been implemented in BlenX (Dematté *et al*, 2010) and simulated by means of the Beta Workbench (Dematte *et al*, 2008). The peculiar characteristics of BlenX allowed us to write a complete model from which, thanks to the modularity of the language, all the different models are obtained by commenting the fragments of code we are not interested in.

The basic metaphor of BlenX is that a biological entity (i.e., a component that is able to interact with other components to accomplish some biological functions) is represented by a computational device called box. A box has a set of interfaces and an internal program and can be graphically represented. Interfaces have associated types and they are used by a box to interact with other boxes; the internal program, instead, codifies for the set of actions that a box can perform after a specific interaction with another box in the system has happened. For example, if a box is modeling a protein, its interfaces may represent sensing and effecting domains.

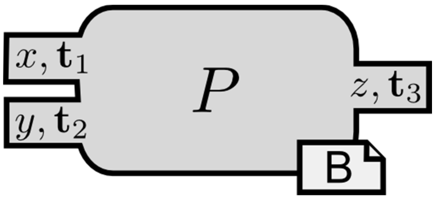

Boxes as abstractions of biological entities. *The small squares on the border of the box are the interfaces;* ***x*** *and* ***y*** *are the interface subjects (omitted when not necessary);* ***t1*** *and* ***t2*** *are the interface types (omitted when not necessary);* ***P*** *is the internal process and* ***B*** *is the name of the box. Subjects are used by the internal program to refer to interfaces.*

Through sensing domains the protein receives signals that are then propagated through the internal actions to activate/inactivate a set of effecting domains. The exchange of signals can happen between boxes whose interfaces have a certain degree of affinity, which codes the strength of their interaction. These affinities are calculated by definable expressions, which can be declared as simple real numbers if the reaction that they are accounting for is an elementary mass action law, or they can be arbitrary functions (e.g., Michaelis-Menten, Hill response,…) if the reaction is an aggregated process whose elementary mechanism of interaction between entities is not known.

We use a small simplified example to introduce briefly the language and the methodology (design pattern) we used to implement our models. Consider two boxes **B1** and **B2** representing, respectively, *activator* and *TR*:

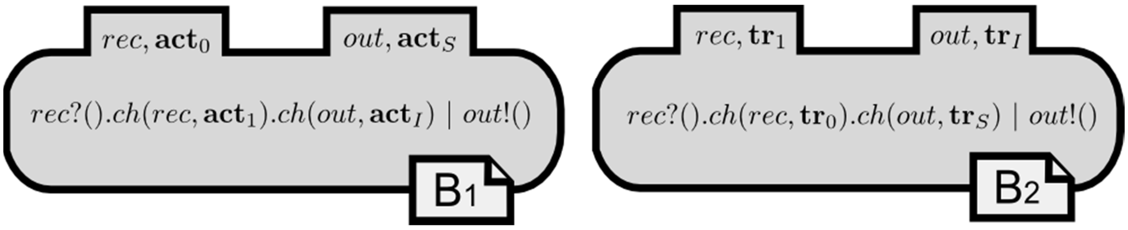

Boxes of activator and TR.

For both boxes interfaces with subject **rec** represent sensing domains, and are used by our proteins to receive activation and inhibition signals. Interfaces with subject out represent effecting domains, and are used by our proteins to send activation and inhibition signals. Types with subscript **0** represent sensing domains of proteins in active forms, while types with subscript **1** represent sensing domains of proteins in inactive forms. Moreover, types with subscript **S** represent effecting domains of proteins in active forms, while types with subscript **I** represent effecting domains of proteins in inactive forms.

Box **B1** can execute a sequence of actions, starting with **rec?()**, in parallel (denoted with **|** symbol) with the action **out!()**. The action **out!()** sends a signal through interface with subject out. The primitive **rec?()** waits a signal on the interaction site with subject **rec** that enables the change of the types of both interfaces by means of the sequence of actions **ch(rec, tr0).ch(out, trS)**. The same holds for box **B2**.

Given the structure of boxes **B1** and **B2**, when types **tr1** and **cdcS** are affine, the two boxes can interact by synchronizing on the corresponding interfaces through the corresponding **out!()** and **rec?()** actions. Specifying the affinity between types **tr1** and **cdcS** as an expression representing the kinetics law used to describe reaction R_2 (see Table S2), we have that the **B1** and **B2** interaction follows the Michaelis-Menten kinetics, causing the transformation of box **B2** into **B2'**, which represents the active protein *TR**:

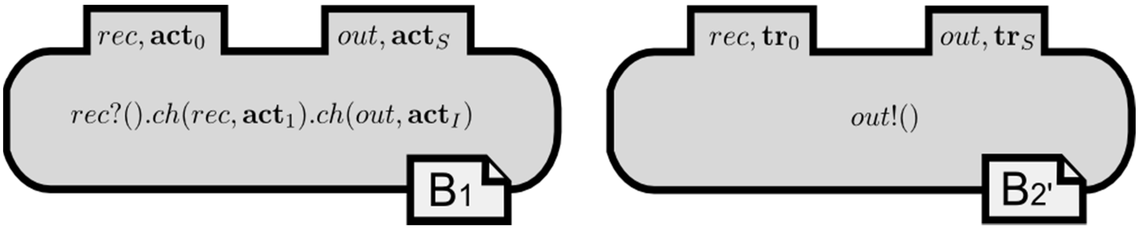

Boxes of activator and TR* after TR activation.

Specifying the affinity between types **trS** and **act0** as an expression representing the kinetics law used to describe reaction R_8 (see Table 1), we have that also **B1** and **B2'** can perform an interaction, which causes the transformation of **B1** in a box that represents the inactive protein *activator*.

This small and simplified example shows how we can implement our protein species as boxes following a common methodology, using simple and reusable internal programs; moreover, it

shows how the ability of a protein to activate or inhibit another protein is specified through the specification of the affinity relation between the types.

Stochastic simulations are performed by means of the Beta Workbench stochastic simulator (Dematte *et al*, 2008), which implements a variant of the Gillespie algorithm (Gillespie, 1977) based on the Gibson and Bruck implementation (Gibson and Bruck, 2000).

A complete and detailed description of BlenX can be found in (Dematté *et al*, 2010), along with a more detailed description of the implementation methodology adopted and here briefly introduced.

4. Simulations of GO and STOP transcriptional regulations

Figure 2 plots simulations of the basic models B_A and B_I, without checkpoint, with positive feedback on both *inhibitor* and *activator*, and with periodic transcription on either *activator* or *inhibitor*, respectively. Transcription factors TF_A and TF_I are activated at time t = 0, together with TF_{TR}. When TFs are present in high amount (top row) then the transitions look qualitatively the same, the threshold of TR^* = 150 is reached approximately after the same time. On the contrary, when TFs are present in low amount (bottom row) then the two models behave totally differently. 1/10 of the normal TF_A level seems to be too weak transcriptional input to induce a transition ever. The same 10 times lower transcription of inhibitor cannot delay the transition and TR^* turns into its active form soon after it is produced. Thus a transcriptional failure of TF_A acts as a STOP signal to the transition, while a failure of TF_I gives a GO signal.

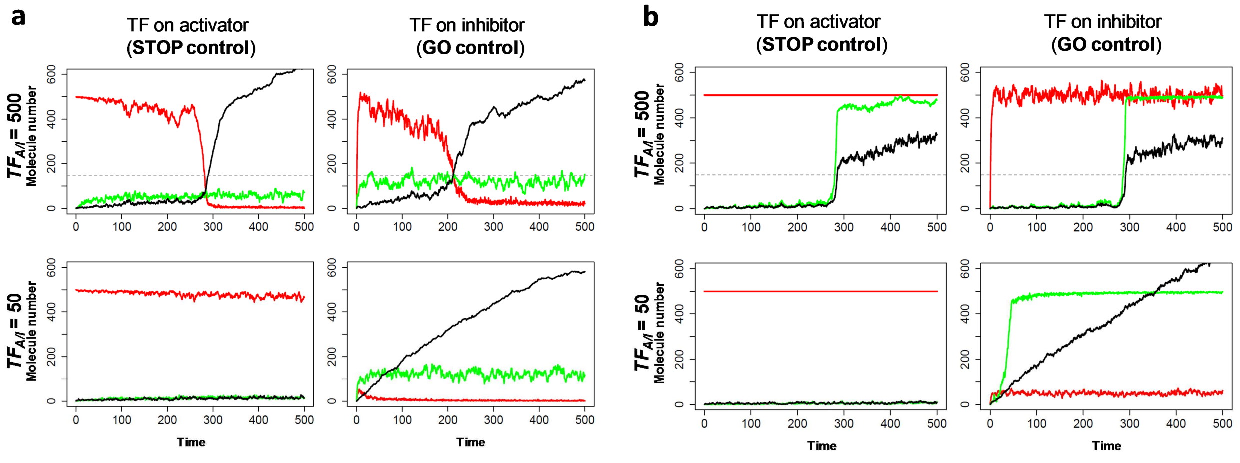

Figure S2: Transcriptional STOP and GO controls with positive feedback only on the inhibitor (a) or only on the activator (b)*. Here we plot the active forms of the key molecules of (a) model* I_A *(right) and* I_I *(left) and (b)* A_A *(right) and* A_I *(left) at high (top) and low (bottom) transcription factor levels. Black: TR*, red: inhibitor*, green; activator**

Similarly, Figure S2a plots simulations of the basic models I_A and I_I. When TFs are present in high amount (top row) then the transitions look qualitatively the same, the threshold of TR^* = 150 is reached approximately after the same time. Note that here the transition is less sharp compared to the one in Figure 2. Although in the figure model I_I tends to reach the threshold earlier, values of S signal (30 and 40 for models I_A and I_I., respectively) have been chosen to

let the two models having the transition in average at approximately the same time. Also in this case when TFs are present in low amount (bottom row) then the two models behave totally differently. Also in this case a transcriptional failure of TF_A acts as a STOP signal to the transition, while a failure of TF_I gives a GO signal.

Finally, Figure S2b plots simulations of the basic models A_A and A_I. Also in these cases simulation results show the same dynamics of the ones reported in Figures 2 and S2a, both when TFs are present in high amount and when TFs are present in low amount. Note however that when the TFs are present in high amount, although the transition is sharp, we have that the maximal value of TR* is lower; indeed, since the inhibitor is always present only in its active form, the system is not able to convert all the TR into TR*.

Worth to notice is the effect of the noise on transcription on the noise in protein levels. In the right panels of Fig S2a,b the inhibitor is under transcriptional control and as a result the inhibitor* level is much noisier. Similar, although less obvious increase in activator* noise can be observed when the activator is under transcriptional regulation. On Fig S2b left panel the inhibitor is not affected neither by transcription or feedback regulation, so we ended up using a constant value for its level.

5. Simulation methods and details on the main figures of the paper

Here we provide details on the different analyses we performed on our models and presented in the figures of the main text.

Robustness of the different models to perturbation in timing of transcriptional induction of activator or inhibitor relative to the transcriptional induction of TR (Figure 3 and Figure S3). We considered model pairs B_A/B_I , I_A/I_I and A_A/A_I ; in red are depicted results of models with TF_I acting on the inhibitor, while in green results of models with TF_A acting on the activator. For each of these models we considered 31 different variants where TFs are assumed to be induced at different timings in the set:

$$\{-50,-45,-40,\ldots,-5,0,5,\ldots,40,45,50,\ldots,90,95,100\}$$

With time 0 we refer to the time point where the transcription of *TR* is initiated (TF_{TR} is set from 0 to 500). For times greater than 0, we used a specific feature of BlenX that allowed us to start simulations with TF_I and TF_A equal to 0 and to change, at the desired time, the amount of TFs from 0 to 500. For times less than 0, we run simulations with initial populations of *activator* and *inhibitor* that reflect the assumption of a TF_A or TF_I induction in advance. In particular, to calculate these initial populations of *activator* and *inhibitor*, we used the formula:

$$\frac{e^{d*t} * \left(s * e^{-d*t} - s\right)}{d} * 500$$

where *s* is the synthesis rate, *d* the degradation rate and *t* is the transcriptional induction time. For each of these 186 models we ran 1000 stochastic simulations and measured, for each simulation, the time at which *TR** reaches population 150 (which value corresponds to the approximate inflection point on the *TR** activation curve, see Figure 4 top panels). We used

the stochastic simulations to calculate means and standard deviations of transition times that are presented by solid lines and background shading, respectively, on Figure 3 and Figure S3.

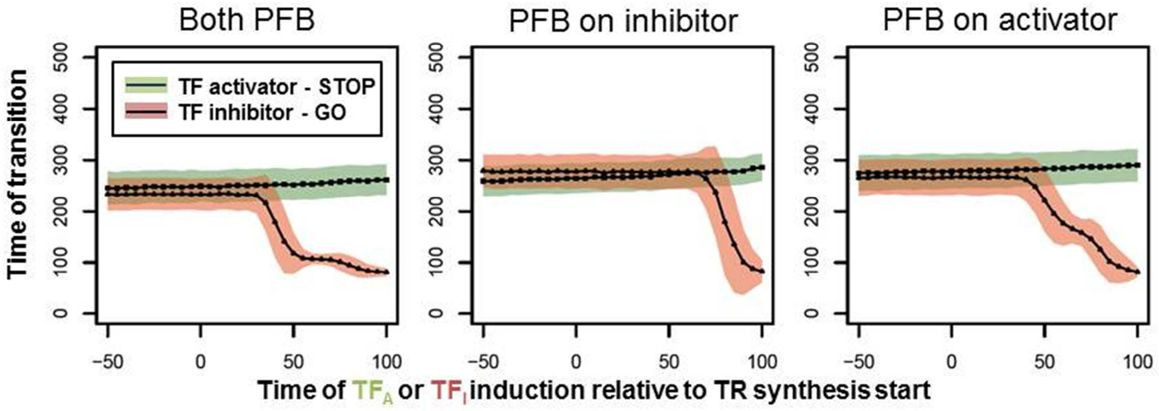

Figure S3: Bistability in cell cycle transitions under various control models*. This figure extends Figure 3 in the main text with all the other considered models.*

Bistability in cell cycle transitions under various control models (Figure 4 and Figure S4). We considered models B_A, B_I, I_A, I_I, A_A and A_I; in red are depicted results of models with *TF* on the inhibitor, while in green results of models with *TF* on the activator. For each of the models we set parameter *kms* to an initial value of 0.0001 and used a specific feature of BlenX that allows to update the value of a parameter when time reaches a certain value. In each single simulation we increased the value of *kms* by adding 0.0001 at times 5000, 10000, 15000, …, 95000 and decreased its value by subtracting 0.0001 at times 100000, 105000, …, 190000. Hence, in a single simulation the value of *kms* is increased at fixed time intervals from value 0.0001 to value 0.002 and then decreased in the same way from value 0.002 to value 0.0001 again.

For each of these 6 models we ran 100 stochastic simulations and for each simulation we calculated in each time interval [(i-1)*5000,i*5000] (with i=1,…,39) the mean values of *TR** inside the sub-intervals [i*5000-2000,i*5000]; we calculated hence the steady state values of *TR** corresponding to the different values of the *kms* parameter.

We then calculated means and standard deviations of these steady state values of TR* over the 100 stochastic simulations and obtained the results reported in Figure 4 and Figure S4.

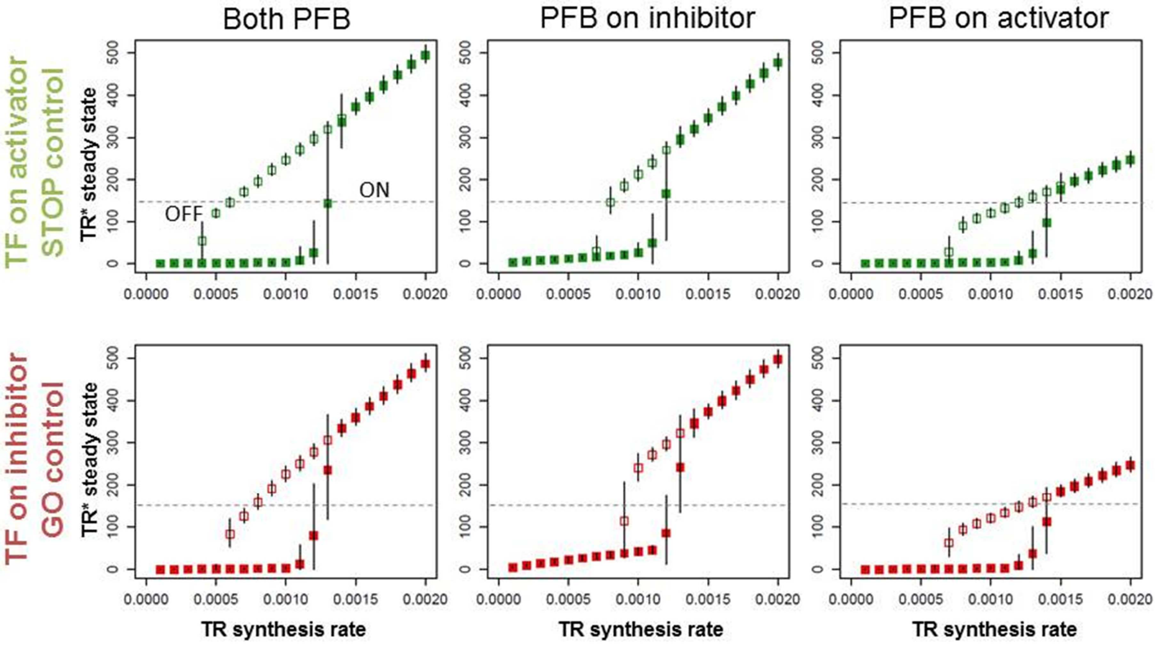

*Figure S4: **Bistability in cell cycle transitions under various control models**. This figure extends Figure 4 in the main text with all the other considered models.*

Parameter robustness test of the various models (Figure 5 and Figure S5). We considered models B_A, B_I, I_A, I_I, A_A and A_I; in red are depicted results of models with *TF* on the inhibitor, while in green results of models with *TF* on the activator. Our robustness analysis is inspired by (Barkai and Leibler, 1997). Each of the six models is considered as a reference model and from each of it we generated 1000 variants by randomly modifying some parameters. For models with *TF* on the activator we modify parameters *kcs*, *kcd*, kcd_1 and for models with *TF* on the inhibitor we modify parameters *kws*, *kwd*, kwd_1. Each alternation of the reference system is characterized by the total parameter variation, *k*, which is defined as:

$$log_{10}(k) = \sum_{n=1}^{L} \left| log_{10} \frac{k_n}{k_n^0} \right|$$

where *L* is the list of the parameters subject to variation, k_n is the altered parameter and k_n^0 is the corresponding parameter in the reference model. Given a parameter k_n^0, the altered parameters are obtained by multiplying k_n^0 to a value *x*, generated using a loguniform distribution in the interval [0.1-10].

For each of the generated 6000 models we ran 100 stochastic simulations and measured, for each simulation, the time at which *TR** reaches population 150. For each combination of each model we calculated the mean (over the 100 simulations) of the times at which *TR** reaches population 150 and plotted results in Figure 5 and Figure S5.

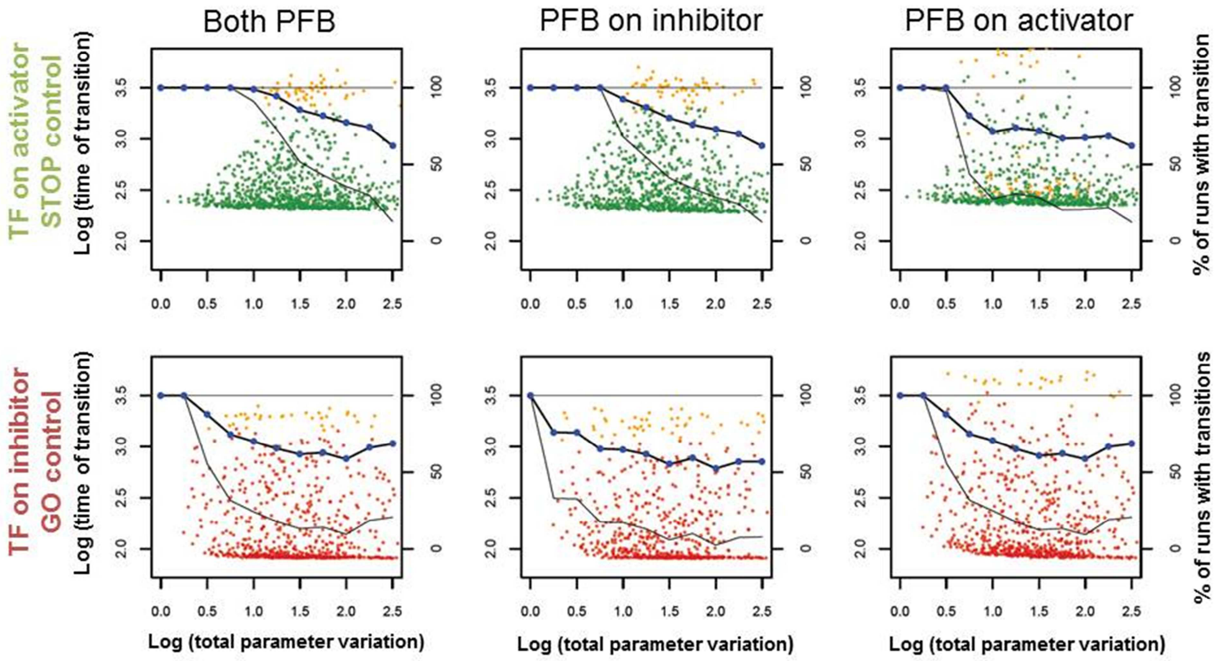

__Figure S5: Parameter robustness test of the various models__. This figure extends Figure 5 in the main text with all the other considered models.

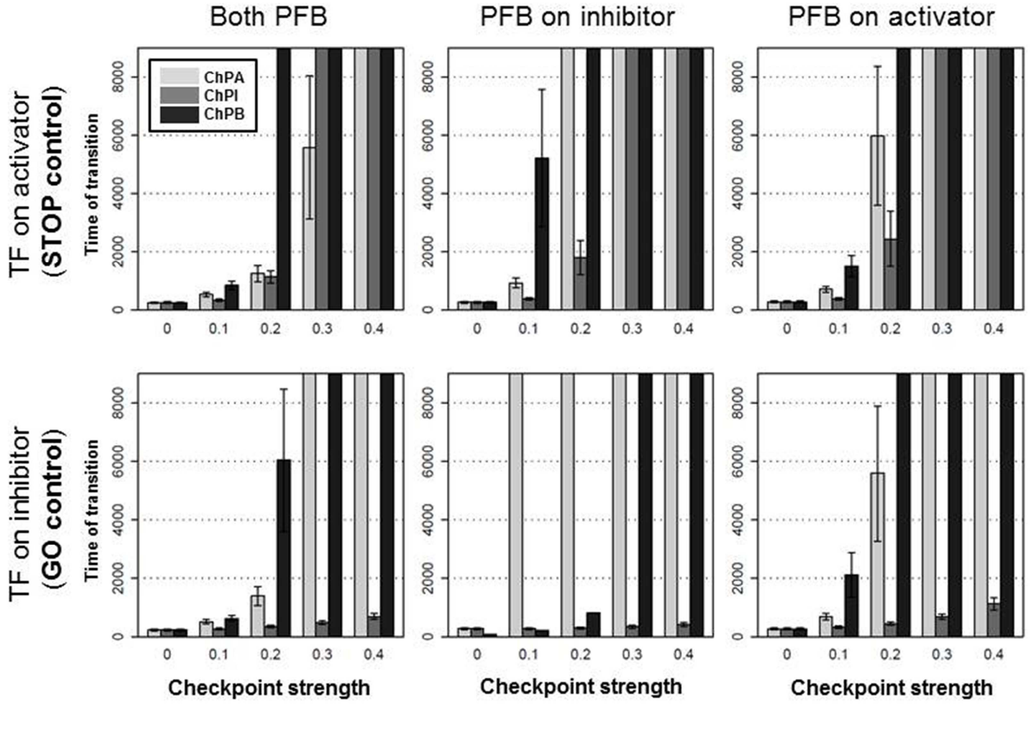

__Figure S6: Checkpoint efficiency on various versions of cell cycle transition control models__. This figure extends Figure 6 in the main text withal the other considered models.

Checkpoint efficiency on various versions of cell cycle transition control models (Figure 6 and Figure S6). We considered pairs of B_I^A/B_A^A, B_I^I/B_A^I, B_I^B/B_A^B, I_I^A/I_A^A, I_I^I/I_A^I, I_I^B/I_A^B A_I^A/A_A^A, A_I^A/A_A^A and A_I^B/A_A^B; also in this case in red are depicted results of models with *TF* on inhibitor, while in green results of models with *TF* on the activator. For each of these models we considered 5 different variants obtained by increasing the activity of the checkpoints

ChP_A and *ChP_I*. In particular, for models with checkpoint on the activator we generated different models with parameter kcp_8 with values in this in the set {0, 0.1, 0.2, 0.3, 0.4}, while for models with checkpoint on the inhibitor we generated different models with parameters kcp_1 and kcp_7 both having value (the same) in the set {0, 0.1, 0.2, 0.3, 0.4}.

For each of the generated 90 models we ran 1000 stochastic simulations and measured, for each simulation, the time at which *TR** reaches population 150 for all models. We used these simulations to calculate means and standard deviations of transition times.

Full bars in the figure indicate that with this parameter value in more than the 90% of the 1000 simulations *TR** do not reach population 150; the star means that the percentage is < 90% but is not 0%. Table S75 shows for all the generated models the exact percentages of simulations (over the 1000) where *TR** reaches population 150.

**CP on activator**						
	Both PBF		PBF on inhibitor		PBF on activator	
Parameter	TF activator	TF inhibitor	TF activator	TF inhibitor	TF activator	TF inhibitor
0	100%	100%	100%	100%	100%	100%
0.1	100%	100%	100%	0%	100%	100%
0.2	100%	100%	25%	0%	22.10%	34.10%
0.3	29.50%	9%	0%	0%	0%	0%
0.4	0%	0%	0%	0%	0%	0%
**CP on inhibitor**						
	Both PBF		PBF on inhibitor		PBF on activator	
Parameter	TF activator	TF inhibitor	TF activator	TF inhibitor	TF activator	TF inhibitor
0	100%	100%	100%	100%	100%	100%
0.1	100%	100%	100%	100%	100%	100%
0.2	100%	100%	100%	100%	100%	100%
0.3	19%	100%	0%	100%	0%	100%
0.4	0%	100%	0%	100%	0%	100%
**CP on both**						
	Both PBF		PBF on inhibitor		PBF on activator	
Parameter	TF activator	TF inhibitor	TF activator	TF inhibitor	TF activator	TF inhibitor
0	100%	100%	100%	100%	100%	100%
0.1	100%	100%	59.80%	100%	100%	100%
0.2	0%	16.40%	0%	100%	0%	0%
0.3	0%	0%	0%	0%	0%	0%
0.4	0%	0%	0%	0%	0%	0%

Table S5: *percentages of simulations where TR* reaches population 150.*

6. Oscillations

We considered models B_A, B_I, I_A, I_I, A_A and A_I. To generate oscillations each of the models is extended with a minimal negative feedback loop model shown below. In the figure *osc1* and *osc1** represent respectively the active and inactive forms of a new intermediary enzyme and *osc2* and *osc2** represent respectively the active and inactive forms of another new intermediary enzyme. In detail, *TR** activates *osc1* that, when active, activates *osc2* which, when active, induces the degradation of *TR** and *TR*. Such combination of positive and

negative feedback loops is supposed to give a robust minimal cell cycle oscillator (Ferrell *et al*, 2011; Pomerening *et al*, 2005).

Kinetic laws and related parameters are shown in Table S6. Reactions R_{30}, R_{31}, R_{34} and R_{35} follow the law of mass action, while the other reactions follow the Michaelis-Menten kinetics. Parameters have been tuned to let enzyme *osc1* to activate fast only when *TR** is far above the bistability threshold and, when *osc2* is active, to let *TR** to stay active for an amount of time that realistically can be enough for a cell to finish mitosis.

For each of the generated 6 models we ran simulations of 200 stochastic cell cycles and measured the period of *TR** oscillations; A period is considered starting when *TR** switches on and reaches population level 150. We used these values, for all the models, to calculate mean and coefficients of variations of the *TR** period (see Table S7). Examples of oscillations for all the considered model are shown in Figure S7.

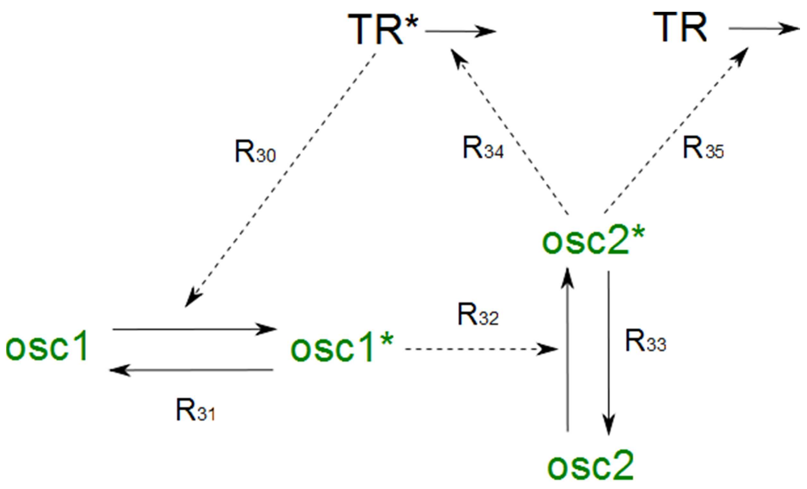

Minimal negative feedback loop model following (Goldbeter, 1991).

$R_{30} = k_1 \times osc1 \times TR^*$	$R_{33} = \frac{k_4 \times osc2^*}{j_4 + (\alpha \times osc2^*)}$	$k_1 = 2e^{-3}$	$j_3 = 5e^{-4}$	$k_1 = 1e^{-4}$
$R_{31} = k_2 \times osc1^*$	$R_{34} = kmd_2 \times osc2^* \times TR^*$	$k_2 = 0.1$	$k_1 = 0.025$	$k_1 = 1e^{-4}$
$R_{32} = \frac{k_3 \times \alpha \times osc1^* \times osc2}{j_3 + (\alpha \times osc2)}$	$R_{35} = kmd_2 \times osc2^* \times TR$	$k_3 = 0.5$	$k_1 = 5e^{-4}$	

Table S6: Kinetic laws and basal parameter set.

	Both PBF (mean/CV)	PBF on inhibitor (mean/CV)	PBF on activator (mean/CV)
TF on activator (STOP)	348.7/9.5	354.9/9.9	351.6/10.1
TF on inhibitor (GO)	316.8/9.9	NA/NA	352.5/11.2

Table S7: Statistic over TR* period.

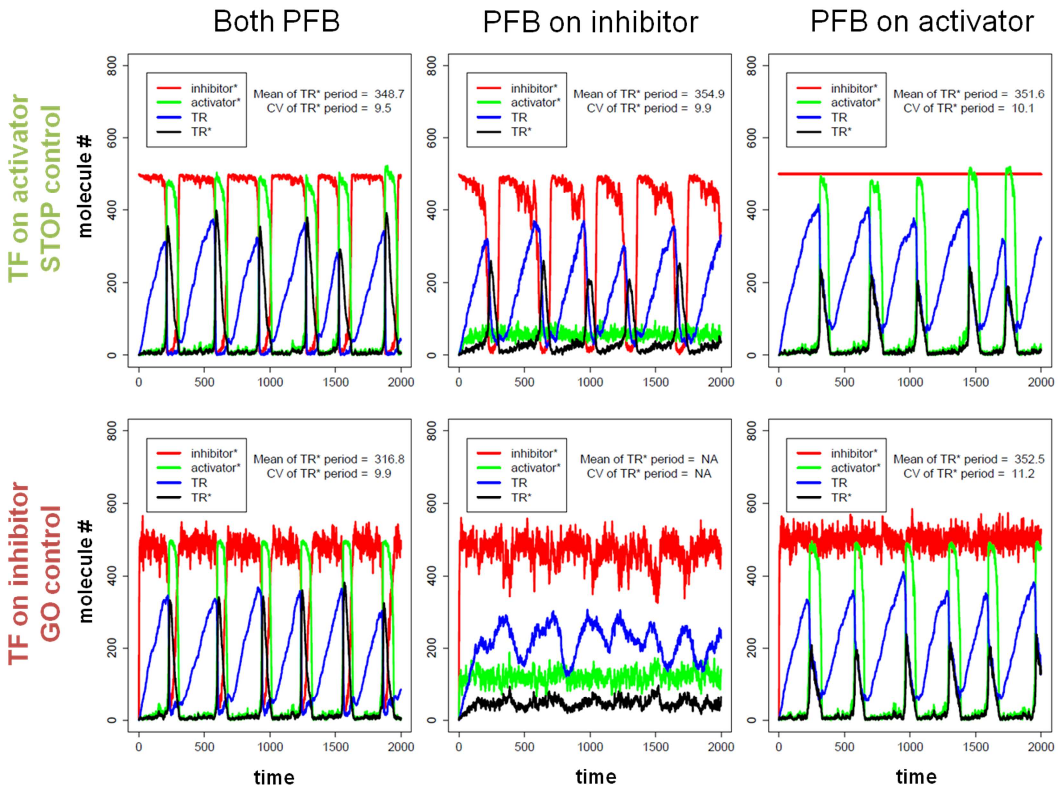

Figure S7: Oscillations. *Oscillations observed for models B_A, B_I, I_A, I_I, A_A and A_I extended with the minimal negative feedback loop model (detailed in Table S6).*

7. Supplementary references

Agarwal R, Cohen-Fix O (2002) Phosphorylation of the mitotic regulator Pds1/securin by Cdc28 is required for efficient nuclear localization of Esp1/separase. *Genes & development* **16:** 1371.

Aligue R, Wu L, Russell P (1997) Regulation of Schizosaccharomyces pombe Wee1 tyrosine kinase. *J Biol Chem* **272:** 13320-13325.

Amon A (1997) Regulation of B-type cyclin proteolysis by Cdc28–associated kinases in budding yeast. *The EMBO Journal* **16:** 2693-2702.

Barkai N, Leibler S (1997) Robustness in simple biochemical networks. *Nature* **387:** 913-917.

Bembenek J, Yu H (2001) Regulation of the Anaphase-promoting Complex by the Dual Specificity Phosphatase Human Cdc14a. *Journal of Biological Chemistry* **276:** 48237-48242.

Bloom J, Cross FR (2007) Novel role for Cdc14 sequestration: Cdc14 dephosphorylates factors that promote DNA replication. *Molecular and Cellular Biology* **27:** 842.

Bollen M, Gerlich DW, Lesage B (2009) Mitotic phosphatases: from entry guards to exit guides. *Trends Cell Biol* **19:** 531-541.

Booher RN, Deshaies RJ, Kirschner MW (1993) Properties of *Saccharomyces cerevisiae* wee1 and its differential regulation of p34^{CDC28} in response to G1 and G2 cyclins. *EMBO J* **12:** 3417-3426.

Boutros R, Dozier C, Ducommun B (2006) The when and wheres of CDC25 phosphatases. *Current opinion in cell biology* **18:** 185-191.

Burgess A, Vigneron S, Brioudes E, LabbÃ© J-C, Lorca T, Castro A (2010) Loss of human Greatwall results in G2 arrest and multiple mitotic defects due to deregulation of the cyclin B-Cdc2/PP2A balance. *Proc Natl Acad Sci U S A* **107:** 12564-12569.

Campbell SD, Sprenger F, Edgar BA, O'Farrellt PH (1995) Drosophila Weel Kinase Rescues Fission Yeast from Mitotic Catastrophe and Phosphorylates Drosophila Cdc2 In Vitro. *Molecular biology of the cell* **6:** 1333-1347.

Chen CT, Feoktistova A, Chen JS, Shim YS, Clifford DM, Gould KL, McCollum D (2008) The SIN kinase Sid2 regulates cytoplasmic retention of the S. pombe Cdc14-like phosphatase Clp1. *Current Biology* **18:** 1594-1599.

Ciliberto A, Capuani F, Tyson JJ (2007) Modeling networks of coupled enzymatic reactions using the total quasi-steady state approximation. *PLoS Comput Biol* **3:** e45.

Ciliberto A, Novak B, Tyson JJ (2003) Mathematical model of the morphogenesis checkpoint in budding yeast. *J Cell Biol* **163:** 1243-1254.

Coleman TR, Dunphy WG (1994) Cdc2 regulatory factors. *Curr Opin Cell Biol* **6:** 877-882.

Costanzo M, Nishikawa JL, Tang X, Millman JS, Schub O, Breitkreuz K, Dewar D, Rupes I, Andrews B, Tyers M (2004) CDK activity antagonizes Whi5, an inhibitor of G1/S transcription in yeast. *Cell* **117:** 899-913.

D'Angiolella V, Palazzo L, Santarpia C, Costanzo V, Grieco D (2007) Role for Non-Proteolytic Control of M-phase Promoting Factor Activity at M-phase Exit. *PloS one* **2**.

de Bruin RAM, McDonald WH, Kalashnikova TI, Yates Iii J, Wittenberg C (2004) Cln3 activates G1-specific transcription via phosphorylation of the SBF bound repressor Whi5. *Cell* **117:** 887-898.

Deibler RW, Kirschner MW (2010) Quantitative reconstitution of mitotic CDK1 activation in somatic cell extracts. *Mol Cell* **37:** 753-767.

Dematté L, Larcher R, Palmisano A, Priami C, Romanel A (2010) Programming Biology in BlenX. In *Systems Biology for Signaling Networks*, Choi S (ed), pp 777-821. Heidelberg: Springer.

Dematte L, Priami C, Romanel A (2008) The Beta Workbench: a computational tool to study the dynamics of biological systems. *Brief Bioinform* **9:** 437-449.

Donzelli M, Draetta GF (2003) Regulating mammalian checkpoints through Cdc25 inactivation. *EMBO Rep* **4:** 671-677.

Durfee T, Becherer K, Chen PL, Yeh SH, Yang Y, Kilburn AE, Lee WH, Elledge SJ (1993) The retinoblastoma protein associates with the protein phosphatase type 1 catalytic subunit. *Genes & development* **7:** 555-569.

Edgar BA, O'Farrell PH (1990) The three postblastoderm cell cycles of *Drosophila* embryogenesis are regulated in G2 by string. *Cell* **62:** 469-480.

Enders GH (2010) Gauchos and ochos: a Wee1-Cdk tango regulating mitotic entry. *Cell Div* **5:** 12.

Esteban V, Blanco M, Cueille N, Simanis V, Moreno S, Bueno A (2004) A role for the Cdc14-family phosphatase Flp1p at the end of the cell cycle in controlling the rapid degradation of the mitotic inducer Cdc25p in fission yeast. *Journal of cell science* **117:** 2461.

Ferrell JE, Tsai TY, Yang Q (2011) Modeling the Cell Cycle: Why Do Certain Circuits Oscillate? *Cell* **144:** 874-885.

Gauthier NP, Jensen LJ, Wernersson R, Brunak S, Jensen TS (2010) Cyclebase.org: version 2.0, an updated comprehensive, multi-species repository of cell cycle experiments and derived analysis results. *Nucleic Acids Res* **38:** D699-702.

Geng Y, Eaton EN, Picon M, Roberts JM, Lundberg AS, Gifford A, Sardet C, Weinberg RA (1996) Regulation of cyclin E transcription by E2Fs and retinoblastoma protein. *Oncogene* **12:** 1173-1180.

Geymonat M, Spanos A, Wells GP, Smerdon SJ, Sedgwick SG (2004) Clb6/Cdc28 and Cdc14 regulate phosphorylation status and cellular localization of Swi6. *Mol Cell Biol* **24:** 2277-2285.

Gibson MA, Bruck J (2000) Efficient exact stochastic simulation of chemical systems with many species and many channels. *J Phys Chem A* **104:** 1876-1889.

Gillespie DT (1977) Exact stochastic simulation of coupled chemical reactions. *J Phys Chem* **81:** 2340-2361.

Goldbeter A (1991) A minimal cascade model for the mitotic oscillator involving cyclin and cdc2 kinase. *Proc Natl Acad Sci U S A* **88:** 9107-9111.

Harvey SL, Charlet A, Haas W, Gygi SP, Kellogg DR (2005) Cdk1-dependent regulation of the mitotic inhibitor Wee1. *Cell* **122:** 407-420.

Hoffmann I, Clarke PR, Marcote MJ, Karsenti E, Draetta G (1993) Phosphorylation and activation of human cdc25-C by cdc2--cyclin B and its involvement in the self-amplification of MPF at mitosis. *EMBO J* **12:** 53-63.

Hoffmann I, Draetta G, Karsenti E (1994) Activation of the phosphatase activity of human cdc25A by a cdk2-cyclin E dependent phosphorylation at the G1/S transition. *EMBO J* **13:** 4302-4310.

Holt LJ, Krutchinsky AN, Morgan DO (2008) Positive feedback sharpens the anaphase switch. *Nature* **454:** 353-357.

Jaspersen SL, Charles JF, Morgan DO (1999) Inhibitory phosphorylation of the APC regulator Hct1 is controlled by the kinase Cdc28 and the phosphatase Cdc14. *Curr Biol* **9:** 227-236.

Kapuy O, He E, Uhlmann F, Novak B (2009) Mitotic exit in mammalian cells. *Mol Syst Biol* **5:** 324.

Karaiskou A, Cayla X, Haccard O, Jessus C, Ozon R (1998) MPF Amplification in Xenopus Oocyte Extracts Depends on a Two-Step Activation of Cdc25 Phosphatase. *Experimental cell research* **244:** 491-500.

Kovelman R, Russell P (1996) Stockpiling of Cdc25 during a DNA replication checkpoint arrest in Schizosaccharomyces pombe. *Mol Cell Biol* **16:** 86-93.

Kramer ER, Scheuringer N, Podtelejnikov AV, Mann M, Peters J-M (2000) Mitotic Regulation of the APC Activator Proteins CDC20 and CDH1. *Mol Biol Cell* **11:** 1555-1569.

Krasinska L, de Bettignies G, Fisher D, Abrieu A, Fesquet D, Morin N (2007) Regulation of multiple cell cycle events by Cdc14 homologues in vertebrates. *Exp Cell Res* **313:** 1225-1239.

Kumagai A, Dunphy WG (1992) Regulation of the cdc25 protein during the cell cycle in Xenopus extracts. *Cell* **70:** 139-151.

Kumagai A, Dunphy WG (1999) Binding of 14-3-3 proteins and nuclear export control the intracellular localization of the mitotic inducer Cdc25. *Genes & development* **13:** 1067.

Lukas J, Lukas C, Bartek J (2004) Mammalian cell cycle checkpoints: signalling pathways and their organization in space and time. *DNA Repair (Amst)* **3:** 997-1007.

Lundberg AS, Weinberg RA (1998) Functional Inactivation of the Retinoblastoma Protein Requires Sequential Modification by at Least Two Distinct Cyclin-cdk Complexes. *Mol Cell Biol* **18:** 753-761.

Mailand N, Falck J, Lukas C, Syljuåsen RG, Welcker M, Bartek J, Lukas J (2000) Rapid destruction of human Cdc25A in response to DNA damage. *Science* **288:** 1425.

Mailand N, Lukas C, Kaiser BK, Jackson PK, Bartek J, Lukas J (2002) Deregulated human Cdc14A phosphatase disrupts centrosome separation and chromosome segregation. *Nature cell biology* **4:** 318-322.

Millar JB, Lenaers G, Russell P (1992) Pyp3 PTPase acts as a mitotic inducer in fission yeast. *The EMBO Journal* **11:** 4933.

Mishra M, Karagiannis J, Trautmann S, Wang H, McCollum D, Balasubramanian MK (2004) The Clp1p/Flp1p phosphatase ensures completion of cytokinesis in response to minor perturbation of the cell division machinery in Schizosaccharomyces pombe. *Journal of cell science* **117:** 3897.

Mueller PR, Coleman TR, Dunphy WG (1995) Cell cycle regulation of a *Xenopus* Wee1-like kinase. *Mol Biol Cell* **6:** 119-134.

Mura I, Csikasz-Nagy A (2008) Stochastic Petri Net extension of a yeast cell cycle model. *J Theor Biol* **254:** 850-860.

Murakami H, Nurse P (2000) DNA replication and damage checkpoints and meiotic cell cycle controls in the fission and budding yeasts. *Biochemical Journal* **349:** 1.

Nash P, Tang X, Orlicky S, Chen Q, Gertler FB, Mendenhall MD, Sicheri F, Pawson T, Tyers M (2001) Multisite phosphorylation of a CDK inhibitor sets a threshold for the onset of DNA replication. *Nature* **414:** 514-521.

O'Connell MJ, Walworth NC, Carr AM (2000) The G2-phase DNA-damage checkpoint. *Trends Cell Biol* **10:** 296-303.

Pal G, Paraz MT, Kellogg DR (2008) Regulation of Mih1/Cdc25 by protein phosphatase 2A and casein kinase 1. *J Cell Biol* **180:** 931-945.

Palou G, Palou R, Guerra-Moreno A, Duch A, Travesa A, Quintana DG (2010) Cyclin regulation by the S phase checkpoint. *Journal of Biological Chemistry*.

Parker LL, Piwnica-Worms H (1992) Inactivation of the p34cdc2-cyclin B complex by the human WEE1 tyrosine kinase. *Science* **257:** 1955-1957.

Pomerening JR, Kim SY, Ferrell JE, Jr. (2005) Systems-level dissection of the cell-cycle oscillator: bypassing positive feedback produces damped oscillations. *Cell* **122:** 565-578.

Potapova TA, Daum JR, Byrd KS, Gorbsky GJ (2009) Fine tuning the cell cycle: activation of the Cdk1 inhibitory phosphorylation pathway during mitotic exit. *Mol Biol Cell* **20:** 1737-1748.

Price D, Rabinovitch S, O'Farrell PH, Campbell SD (2000) Drosophila wee1 has an essential role in the nuclear divisions of early embryogenesis. *Genetics* **155:** 159.

Queralt E, Uhlmann F (2008) Cdk-counteracting phosphatases unlock mitotic exit. *Current opinion in cell biology* **20:** 661-668.

Rhind N, Russell P (1998) Mitotic DNA damage and replication checkpoints in yeast. *Curr Opin Cell Biol* **10:** 749-758.

Russell P, Nurse P (1986) $cdc25^{+}$ functions as an inducer in the mitotic control of fission yeast. *Cell* **45:** 145-153.

Russell P, Nurse P (1987) Negative regulation of mitosis by wee1+, a gene encoding a protein kinase homolog. *Cell* **49:** 559-567.

Sanchez Y, Wong C, Thoma RS, Richman R, Wu Z, Piwnica-Worms H, Elledge SJ (1997) Conservation of the Chk1 checkpoint pathway in mammals: linkage of DNA damage to Cdk regulation through Cdc25. *Science* **277:** 1497-1501.

Schwob E, Nasmyth K (1993) CLB5 and CLB6, a new pair of B cyclins involved in DNA replication in Saccharomyces cerevisiae. *Genes & development* **7:** 1160.

Sia RA, Bardes ES, Lew DJ (1998) Control of Swe1p degradation by the morphogenesis checkpoint. *EMBO J* **17:** 6678-6688.

Sibon OCM, Stevenson VA, Theurkauf WE (1997) DNA-replication checkpoint control at the Drosophila midblastula transition. *Nature* **388:** 93-96.

Stanford JS, Ruderman JV (2005) Changes in regulatory phosphorylation of Cdc25C Ser287 and Wee1 Ser549 during normal cell cycle progression and checkpoint arrests. *Mol Biol Cell* **16:** 5749-5760.

Stumpff J, Duncan T, Homola E, Campbell SD, Su TT (2004) Drosophila Wee1 kinase regulates Cdk1 and mitotic entry during embryogenesis. *Curr Biol* **14:** 2143-2148.

Sveiczer A, Novak B, Mitchison JM (1999) Mitotic control in the absence of cdc25 mitotic inducer in fission yeast. *Journal of cell science* **112:** 1085.

Taberner FJ, Quilis I, Igual JC (2009) Spatial regulation of the start repressor Whi5. *Cell Cycle* **8:** 3010-3018.

Tyers M, Futcher B (1993) Far1 and Fus3 link the mating pheromone signal transduction pathway to three G1-phase Cdc28 kinase complexes. *Mol Cell Biol* **13:** 5659-5669.

Visintin R, Craig K, Hwang ES, Prinz S, Tyers M, Amon A (1998) The phosphatase Cdc14 triggers mitotic exit by reversal of Cdk-dependent phosphorylation. *Molecular cell* **2:** 709-718.

Visintin R, Prinz S, Amon A (1997) CDC20 and CDH1: a family of substrate-specific activators of APC-dependent proteolysis. *Science* **278:** 460.

Wade Harper J, Adami GR, Wei N, Keyomarsi K, Elledge SJ (1993) The p21 Cdk-interacting protein Cip1 is a potent inhibitor of G1 cyclin-dependent kinases. *Cell* **75:** 805-816.

Watanabe N, Arai H, Iwasaki J, Shiina M, Ogata K, Hunter T, Osada H (2005) Cyclin-dependent kinase (CDK) phosphorylation destabilizes somatic Wee1 via multiple pathways. *Proc Natl Acad Sci U S A* **102:** 11663-11668.

Watanabe N, Broome M, Hunter T (1995) Regulation of the human WEE1Hu CDK tyrosine 15-kinase during the cell cycle. *EMBO J* **14:** 1878-1891.

Wolfe BA, Gould KL (2004) Fission yeast Clp1p phosphatase affects G2/M transition and mitotic exit through Cdc25p inactivation. *The EMBO Journal* **23:** 919-929.

Wolfe BA, McDonald WH, Yates Iii JR, Gould KL (2006) Phospho-regulation of the Cdc14/Clp1 phosphatase delays late mitotic events in S. pombe. *Developmental cell* **11:** 423-430.

Yoshida S, Asakawa K, Toh-e A (2002) Mitotic exit network controls the localization of Cdc14 to the spindle pole body in Saccharomyces cerevisiae. *Current Biology* **12:** 944-950.

Zachariae W, Schwab M, Nasmyth K, Seufert W (1998) Control of cyclin ubiquitination by CDK-regulated binding of Hct1 to the anaphase promoting complex. *Science* **282:** 1721.

Zarzov P, Decottignies A, Baldacci G, Nurse P (2002) G(1)/S CDK is inhibited to restrain mitotic onset when DNA replication is blocked in fission yeast. *EMBO J* **21:** 3370-3376.
